# Efficacy and Safety of Insulin Glargine 300 U/mL versus 100 U/mL in Diabetes Mellitus: A Comprehensive Review of the Literature

**DOI:** 10.1155/2018/2052101

**Published:** 2018-02-12

**Authors:** Hernando Vargas-Uricoechea

**Affiliations:** Metabolic Diseases Study Group, Division of Endocrinology and Metabolism, Department of Internal Medicine, Universidad del Cauca, Popayán, Cauca, Colombia

## Abstract

To achieve good metabolic control in diabetes and maintain it in the long term, a combination of changes in lifestyle and pharmacological treatment is necessary. The need for insulin depends upon the balance between insulin secretion and insulin resistance. Insulin is considered the most effective glucose-lowering therapy available and is required by people with type 1 diabetes mellitus to control their blood glucose levels; yet, many people with type 2 diabetes mellitus will also eventually require insulin therapy, due to the progressive nature of the disease. A variety of long-acting insulins is currently used for basal insulin therapy (such as insulin glargine, degludec, and detemir), each having sufficient pharmacodynamic and pharmacokinetic profiles to afford lower intrapatient variability and an extended duration of action. The new glargine-300 formulation was developed to have a flatter and more extended time-action profile than the original glargine-100, and these characteristics may translate into more stable and sustained glycemic control over a 24 h dosing interval. The objective of this comprehensive review was to summarize the available evidence on the clinical efficacy and safety of glargine-300 versus glargine-100 from the EDITION clinical trial program, in patients with type 1 and type 2 diabetes mellitus.

## 1. Introduction

The frequency of diabetes mellitus (DM) has increased worldwide, leading to a huge social, economic, and healthcare burden. DM is considered one of the diseases leading to major healthcare changes in every country, regardless of the income level or socioeconomic status. As of 2016, the World Health Organization has estimated that over 422 million adults were living with DM in 2014, compared to 108 million in 1980. The global prevalence (age-standardized) of DM has nearly doubled since 1980, rising from 4.7% to 8.5% in the adult population. This phenomenon is due to the increase in associated risk factors, such as overweight or obesity [1, 2]. According to the International Diabetes Federation, 415 million people worldwide, or 8.8% of adults aged 20–79, are estimated to have diabetes, and about 75% live in low- and middle-income countries. If these trends continue, by 2040, some 642 million people, or 1 adult in every 10, will have diabetes [3, 4].

DM is a chronic disease characterized by hyperglycemia from defects in insulin secretion, insulin action, or both. Type 1 (T1) DM usually begins at a young age and is mainly due to autoimmune destruction of the pancreatic *β*-cells; as a result, patients with T1DM require lifelong insulin supplementation for survival [5, 6]. In type 2 (T2) DM, the combined impact of impaired insulin secretion and insulin resistance results in elevated blood glucose (BG) levels. In T2DM, the treatment initially involves changes in lifestyle (diet, exercise), with oral antihyperglycemic drugs (OADs) added as necessary to maintain adequate BG control [7, 8].

Other injectable therapeutic agents, such as the glucagon-like peptide-1 (GLP-1) receptor agonists, may be an option before insulin therapy, or in addition to insulin in some patients. The number of patients requiring insulin is expected to rise more steeply. Insulin may be the desired therapy in individuals with T2DM with critical beta-cell failure and intolerance to or failure of OADs or due to patient preference. In T1DM, basal insulin in combination with rapid-acting mealtime insulin provides an adequate but imperfect replacement for endogenous physiologic insulin production [9, 10].

The purpose of this review was to assess the relevant available evidence of the efficacy, safety, and clinical applicability to evaluate insulin glargine 300 U/mL (Glar-300).

## 2. Data Selection

The following electronic databases were searched: PubMed and MEDLINE (using the Ovid platform), Scopus, BIOSIS, Embase, ClinicalTrials.gov, Google Scholar, and Springer Online Archives Collection, from January 1966 to July 2017, using the terms “insulin,” “glargine,” “glargine 300,” “glargine 100,” and “basal insulin” in combination with the term “diabetes.” Articles resulting from these searches and relevant references cited in those articles were examined. International conference proceedings on DM (2016-2017) were also reviewed. In addition, a manual search of some reference lists of relevant reviews and trials was performed. Only articles published in English were included.

## 3. General Considerations about the Clinical Results with Analogue Basal Insulins

Long-acting insulin analogues such as glargine (Glar), detemir (Det), and degludec (Deg) were developed to mimic the peakless and continuous kinetic profile of physiologic basal insulin secretion. They are aimed at overcoming some of the consequences resulting from the clinical use of neutral protamine Hagedorn (NPH) insulin (i.e., absorption variability and risk of hypoglycemia). Considerable progress has been made in terms of pharmacokinetics (PKs) and pharmacodynamics (PDs), including longer action, sustained glucose-lowering effect (with lower risk of hypoglycemia), low intraindividual variability, and potential higher flexibility of the administration regimes [11, 12]. Consequently, the clinical trials involving T2DM (insulin-naïve) patients, comparing Glar 100 U/mL (Glar-100) and NPH (and the concomitant use of OADs), showed similar metabolic control in terms of hemoglobin (Hb) A1c and fasting plasma glucose (FPG), regardless of the OAD-based management, with lower rates of global and nocturnal hypoglycemia in the Glar-100-treated patients. Furthermore, there was a slight increase in the total daily dose of insulin among the Glar-100 group, as well as an increase in weight gain, particularly among those receiving sulphonylureas (SUs) [13, 14]. Studies in T1DM adults showed that management with Glar-100 resulted in a mild but significant decrease in the level of HbA1c, with less episodes of severe hypoglycemia and nocturnal hypoglycemia, as compared to NPH management [15–17].

Moreover, clinical trials comparing Det insulin and NPH in T2DM failed to recognize any differences in the HbA1c level (in individuals receiving combined management with insulin bolus or OADs). Additionally, a higher proportion of individuals achieved the HbA1c < 7% goal, with lower rates of confirmed hypoglycemia with Det. Patients managed with Det also experienced less weight gain compared to patients receiving NPH [18–21]. Studies in T1DM comparing both insulins showed that Det treatment significantly lowered the HbA1c levels and FPG, with less weight gain and lower risk of hypoglycemia, as compared to NPH management. However, subjects in the Det group required a higher average daily dose than those treated with NPH [22–25].

The clinical trials comparing Det regimes and Glar-100 in T2DM insulin-naïve subjects, or in subjects using the basal-bolus regime, failed to identify any significant differences in terms of the primary goal of achieving the specified HbA1c or FPG levels, or in the proportion of patients achieving the HbA1c goal without symptomatic hypoglycemia. Furthermore, a small but significant weight gain was documented in the population treated with Glar-100; however, at the end of the treatment, the results showed that Det-treated subjects required a higher daily insulin dose and a higher probability of needing to split the daily dose (twice a day) as compared to Glar-100-treated subjects [26–28]. On the other hand, in T1DM patients, the comparison between both insulins showed the noninferiority of Det versus Glar-100 to reach the HbA1c < 7% goal, as well as the FPG values or the proportion of individuals achieving the HbA1c < 7% goal in the absence of confirmed hypoglycemia, although self-measured FPG levels were significantly lower with Glar-100 than with Det. No significant differences were found in terms of weight gain, though the Det-treated patients required a higher average daily dose of insulin than the patients receiving Glar-100 [29, 30].

Finally, the clinical trials comparing Glar-100 and Deg have shown that Deg management provides glycemic control similar to that achieved with Glar-100, with lower risk of nocturnal hypoglycemia and overall hypoglycemia in T2DM patients using the basal-bolus regime. By contrast, in patients treated with basal regimes combined with OADs, the use of Deg was associated with lower risk of nocturnal hypoglycemia or severe hypoglycemia, as compared with that of Glar-100. Additionally, the rates of treatment-emergent adverse events (AEs) were similar for both insulins, with no differences in the rates of adverse cardiovascular events; among patients with T2DM at high risk for cardiovascular events, Deg was noninferior to Glar-100 with respect to the incidence of major cardiovascular events [31–33]. Recently, two clinical trials showed that, among patients with T1DM and in T2DM treated with insulin (and with at least one hypoglycemia risk factor), the Deg treatment resulted in a reduced rate of overall symptomatic hypoglycemia, as compared to Glar-100 [34, 35].

## 4. General Observations about GLAR-300

Glar-300 was developed to address the duration concerns of the early basal analogues. Like Glar-100, Glar-300 contains insulin Glar, a 21A-Gly modified mimic of the final intermediate of natural human insulin. Glar-300 has one-third the injection volume of Glar-100 and offers a more even and prolonged PK/PD profile that lasts beyond 24 h. To increase the concentration from 100 to 300 U/mL (following subcutaneous administration) under usual conditions at physiological pH, insulin Glar normally precipitates and aggregates, leading to the formation of a subcutaneous depot from which insulin is subsequently released. The size of the depot precipitate is dependent upon the concentration of the injection solution, while the unit amount remains the same, so that Glar-300 forms a smaller precipitate than Glar-100 [36].

Since the release of Glar molecules from the depot is proportional to the surface area, the smaller precipitate formed with Glar-300 leads to the release of less unit (denoted as U) amounts of Glar over time for a longer period than the larger precipitate that is formed with Glar-100. Consequently, Glar-300 provides more stable glucose levels throughout the day, with low diurnal fluctuation, low intrasubject glucose variability, and high level of between-day reproducibility compared with Glar-100. The less pronounced peak of action could theoretically result in a more gradual reduction in BG, with a reduced risk of hypoglycemia, while achieving glycemic control. Finally, the potency of Glar-300 is stated in units; these units are exclusive to Glar-300 and are different from international units (denoted as IU) or the units used to express the potency of other insulin analogues.

Glar-300 is not bioequivalent to Glar-100, and dose adjustment is needed when patients are switched from Glar-100 or other basal insulins to Glar-300 or vice versa [37, 38]. Glar-300 was approved in early 2015 by both the United States Food and Drug Administration and the European Medicines Agency.

## 5. General Characteristics of the EDITION Trials

The therapeutic efficacy of Glar-300 in patients with DM was examined in several 6 mo, centrally randomized, controlled, open-label, parallel-group, treat-to-target, multinational, multicenter studies involving the phase 3 EDITION trial program. The studies utilized a common core protocol that standardized most aspects of the study design, including the comparator (Glar-100), 1 : 1 randomization, stratification by screening HbA1c, targets for fasting prebreakfast self-monitored plasma glucose (SMPG), recommendations for dosing of Glar-300 and Glar-100, and primary and secondary efficacy variables and safety variables, such as the definitions used for the hypoglycemia categories and analyses [39]. Additionally, patients already on Glar-100 prior to the study were switched over to the study medications at their current dose or, if they had been on NPH or Det, then at 80% of those doses (consistent with the standard recommendation). The target range for median preprandial SMPG for the T1DM patients was 80–130 mg/dL (4.4–7.2 mmol/L), and the target for fasting prebreakfast SMPG in the T2DM studies was 80–100 mg/dL (4.4–5.6 mmol/L). Moreover, the choice of an open-label design (with no blinding of either investigators or participants) was dictated by the need for dose adjustment and the difference between the test and reference formulations in terms of concentration and volume of injection per unit of insulin (i.e., differences in the pen injector devices and volumes in the Glar-300 and Glar-100 treatment groups) [40–44]. The baseline (BL) demographics and patient characteristics of participants in the EDITION clinical trial program are shown in Table 1.

### 5.1. EDITION 1 Trial

The EDITION 1 trial was a 6 mo, multicenter, randomized, open-label, parallel-group trial comparing Glar-300 to Glar-100 while maintaining mealtime insulin, with a 6 mo comparative safety extension period and a follow-up on-site visit at 4 wk posttreatment. Eight hundred and seven participants were randomized to the Glar-300 (*n* = 404) or Glar-100 (*n* = 403) groups, 404 and 402, respectively, received the study insulin (safety population), and 404 and 400 represented the modified intention-to-treat (mITT) population. Treatment was discontinued before 6 mo by 30 (7.4%) of the 404 participants in the Glar-300 group and by 31 (7.7%) of the 403 in the Glar-100 group.

The inclusion criteria were a diagnosis of T2DM and the use of basal and mealtime insulin therapy, including current basal therapy with ≥42 U/d of either Glar-100 or NPH, together with mealtime therapy with insulin lispro, aspart, or glulisine, with or without metformin, for at least 1 year. The exclusion criteria were as follows: age < 18 years; HbA1c < 7.0% or >10% at screening; diabetes other than T2DM; less than 1 year on basal plus mealtime insulin and SMPG; any contraindication to use insulin Glar as defined in the national product label; use of human regular insulin as mealtime insulin in the last 3 mo before the screening visit; use of an insulin pump in the last 6 mo before the screening visit; initiation of new glucose-lowering agents and/or weight loss drugs in the last 3 mo before the screening visit; history or presence of significant diabetic retinopathy or macular edema likely to require laser, injectable drugs, or surgical treatment during the study period; or pregnancy or breast-feeding or intention (for women) to become pregnant during the study period.

The insulin dose was titrated to achieve an FPG of 80–100 mg/dL (4.4–5.6 mmol/L) over an initial titration phase. In a planned extension of this trial, the authors examined whether the pattern of glycemic control, tolerability, and risk of nocturnal hypoglycemia was achieved with continued use of Glar-300 for a further 6 mo interval of randomized but less intensively supervised treatment. At the end of the extension phase, the following efficacy outcomes were assessed: change from BL in glycemic control (HbA1c, FPG, and 8-point SMPG profiles), mean insulin dose (basal and mealtime), and score on the Diabetes Treatment Satisfaction Questionnaire (DTSQ). The DTSQ addresses the participant's satisfaction with the treatment (six items), perceived hyperglycemia (one item), and perceived hypoglycemia (one item), as well as the change from BL in body weight, percentage of participants experiencing ≥1 hypoglycemic event, annualized rates of hypoglycemic events, and occurrence of other AEs [45, 46].

### 5.2. EDITION 2 Trial

The EDITION 2 trial was a 6 mo, multicenter, randomized, open-label, parallel-group study comparing Glar-300 and Glar-100 both plus OAD in patients with T2DM, with a 6 mo safety extension period. A total of 811 participants were randomized to the Glar-300 (*n* = 404) or Glar-100 (*n* = 407) groups. One participant in each group did not receive treatment, and one participant in the Glar-100 group had no BL or post-BL HbA1c measurements; therefore, 403 and 405 participants, respectively, formed the mITT population. Treatment was discontinued by 36 participants (8.9%) in the Glar-300 group and by 38 (9.3%) in the Glar-100 group. The inclusion criterion was diagnosis of T2DM. The exclusion criteria were as follows: age < 18 years, HbA1c < 7.0% or >10% at screening, diabetes other than T2DM, or <6 mo on basal insulin treatment together with OADs and SMPG. Patients who had taken a stable dose of OAD background therapy for 3 mo were eligible to enroll, except for those who had taken SUs, which were prohibited within 2 mo before the screening visit and during participation in the study.

The doses and combinations of OADs were in accordance with the authorized local labeling and were kept stable throughout the study unless any of the following conditions existed: a specific safety issue related to these treatments; use of an insulin pump in the last 6 mo before screening; history or presence of significant diabetic retinopathy or macular edema likely to require laser or injectable drugs or surgical treatment during the study period; or pregnancy or breast-feeding or intention to become pregnant (for women) during the study period. As in EDITION 1, high-dose insulin use was an eligibility criterion in EDITION 2, with participants required to use ≥42 U of basal insulin per day.

After the main 6 mo treatment period, participants in this trial continued in a 6 mo safety extension to examine the longer-term outcomes of treatment with Glar-300 and Glar-100. The authors evaluated the changes in glycemic control (HbA1c, FPG, and SMPG); basal insulin dose from BL to the end of the 12 mo treatment; changes in body weight, status, title, and cross-reactivity with human insulin of anti-insulin antibodies (AIAs); and AEs. Hypoglycemic events at any time (24 h) and during the night, scores from the DTSQ, and a more stringent plasma glucose threshold of <3.0 mmol/L (<54 mg/dL) were also used [47, 48].

### 5.3. EDITION 3 Trial

The EDITION 3 trial was a 6 mo, multicenter, multinational, randomized, two-arm parallel-group, open-label study, comparing the efficacy and safety of Glar-300 and Glar-100 in insulin-naïve patients with T2DM not adequately controlled with noninsulin antihyperglycemic drugs, with a 6 mo safety extension period. Of 878 participants randomized to Glar-300 (*n* = 439) or Glar-100 (*n* = 439), 435 and 438, respectively, received treatment and comprised the safety population; the mITT population comprised 432 and 430 participants, respectively. Treatment was discontinued by 62 (14%) and 75 (17%) participants in the Glar-300 and Glar-100 groups, respectively. Randomization of patients to the Glar-300 or Glar-100 groups was stratified according to HbA1c values at screening (<8.0% or ≥8.0%) and the geographical region (non-Japanese or Japanese), with a minimum of 20% randomized patients per HbA1c stratum.

The inclusion criteria were adults with T2DM inadequately controlled with noninsulin antihyperglycemic drugs. The exclusion criteria were as follows: HbA1c < 7.0% or >11%; history of T2DM for <1 year before screening; <6 mo before screening with OAD treatment; change in dose of OAD treatment in the last 3 mo before screening; initiation of new glucose-lowering medications and/or weight loss drug in the last 3 mo before the screening visit and/or initiation of the GLP-1 receptor agonist in the last 6 mo before the screening visit; current or previous insulin use, except for a maximum of 8 consecutive days (e.g., acute illness and surgery) during the last year prior to screening; and unstable proliferative diabetic retinopathy or any other rapidly progressive diabetic retinopathy or macular edema likely to require treatment during the study period. Participants receiving only noninsulin antihyperglycemic drugs not approved for combination with insulin according to the local labeling/local treatment guidelines and/or SU or glinide were required to be discontinued at BL.

Participants who completed the 6 mo treatment period continued to receive either Glar-300 or Glar-100, according to initial randomization, for a further predefined 6 mo extension phase. The authors evaluated the change from BL to month 12 in HbA1c, FPG, prebreakfast SMPG, 8-point SMPG profiles, and basal insulin dose. Safety/tolerability outcomes included risk of hypoglycemia, change from BL to month 12 in body weight, and the occurrence of other AEs. An additional post hoc exploratory analysis was made according to prior SU use (within the 3 mo period prior to screening or within the run-in period). Bicomposite efficacy endpoints (post hoc, exploratory) were also assessed, defined as the percentage of participants achieving HbA1c target (<7.0%) at month 12 without hypoglycemia (confirmed or severe or documented symptomatic) at night and at any time of the day (24 h) over 12 mo of treatment [49, 50].

### 5.4. EDITION 4 Trial

The EDITION 4 trial was a 6 mo, multicenter, randomized, open-label, parallel-group study comparing the efficacy and safety of Glar-300 and Glar-100 [randomized (1 : 1 : 1 : 1) to once-daily Glar-300 or Glar-100], injected in the morning or evening, while continuing mealtime insulin in patients with T1DM, with a 6 mo safety extension period. A total of 549 people with T1DM were screened, with 274 randomized to Glar-300 and 275 to Glar-100 groups; all received treatment and thus formed the safety population.

The inclusion criteria were adult participants with T1DM. The exclusion criteria were as follows: HbA1c < 7.0% or >10% at screening; less than 1 year on any basal plus mealtime insulin and self-monitoring of BG before the screening visit; unstable insulin dosing (±20 percent total basal insulin dose) in the last 30 d prior to the screening visit; use of premix insulin, human regular insulin as mealtime insulin, and/or any glucose-lowering drugs other than basal insulin and mealtime analogue insulin in the last 3 mo before the screening visit; use of an insulin pump in the last 6 mo before the screening visit and no plan to switch to an insulin pump in the next 12 mo; unwillingness to inject insulin Glar as assigned by the randomization process once daily in the morning or evening; severe hypoglycemia resulting in coma/seizures and/or hospitalization for diabetic ketoacidosis in the last 6 mo before the screening visit; or unstable proliferative diabetic retinopathy or any other rapidly progressive diabetic retinopathy or macular edema likely to require treatment (e.g., laser, surgical treatment, or injectable drugs) during the study period.

Participants who completed the 6 mo main study period continued open-label to take Glar-300 or Glar-100 once daily in the morning or evening (as previously randomized) for a further 6 mo period. The authors evaluated the change in HbA1c from BL to month 12, central laboratory-measured FPG, prebreakfast SMPG, 8-point SMPG profiles, and insulin dose (basal and mealtime), as well as hypoglycemic events, changes in body weight, AEs, and participant-reported satisfaction with the treatment and perception of the occurrence of hypo- and hyperglycemia [using the DTSQ, health-related quality of life (EQ-5D) utility index score, and hypoglycemia fear survey (HFS II)] [51, 52].

### 5.5. EDITION JP1 Trial

The EDITION JP1 was a 6 mo, multicenter, randomized, open-label, parallel-group study comparing the efficacy and safety of Glar-300 and Glar-100 in Japanese people with T1DM who were taking prior basal and mealtime insulin, with a 6 mo extension period. Eligible participants were randomized to Glar-300 (*n* = 122) or Glar-100 (*n* = 121) groups. All randomized participants received study treatment. The discontinuation rate was 4.1% for the Glar-300 group and 3.3% for the Glar-100 group.

The inclusion criterion was diagnosis of T1DM. The exclusion criteria were as follows: age < 18 years at the screening visit; HbA1c < 7.0% or >10.0% at the screening visit; <1 year before the screening visit on any basal plus mealtime insulin; unstable insulin dosing (±20% total basal insulin dose) in the last 30 d prior to the screening visit; use of premix insulin, human regular insulin as mealtime insulin, and/or any glucose-lowering drugs other than basal insulin and mealtime rapid insulin analogue in the last 3 mo before the screening visit; use of an insulin pump in the last 6 mo before the screening visit and/or plan to switch to an insulin pump in the next 12 mo; severe hypoglycemia resulting in coma/seizures and/or hospitalization for diabetic ketoacidosis in the last 6 mo before the screening visit; or unstable proliferative diabetic retinopathy or any other rapidly progressive diabetic retinopathy or macular edema likely to require treatment during the study period [53, 54].

### 5.6. EDITION JP2 Trial

The EDITION JP2 trial was a 6 mo, multicenter, randomized, open-label, parallel-group study comparing the efficacy and safety of Glar-300 and Glar-100 both in combination with OADs in Japanese patients with T2DM, with a 6 mo safety extension period. Of 259 individuals screened, 241 were randomized to Glar-300 (*n* = 121) or Glar-100 (*n* = 120) groups. One participant left the Glar-300 group before receiving any study treatment; the remaining 240 participants were included in the mITT and safety populations.

The inclusion criteria were diagnosis of T2DM for at least 1 year at the time of the screening visit treated with basal insulin in combination with OADs for at least 6 mo before the screening visit. The exclusion criteria were as follows: age < 18 years at the screening visit; body mass index (BMI) ≥35 kg/m^2^ at the screening visit; HbA1c < 7.0% or >10% at the screening visit; diabetes other than T2DM; patients on SMPG <6 mo before the screening visit; use of premix insulin, Det 2 times or more a day, or GLP-1 receptor agonists in the last 3 mo before the screening visit; use of mealtime insulin (rapid-acting insulin analogue and short-acting insulin) for more than 10 d in the last 3 mo before the screening visit; use of an insulin pump in the last 6 mo before the screening visit; initiation of new glucose-lowering medications and/or weight loss drugs in the last 3 mo before the screening visit; severe hypoglycemia resulting in coma/seizures and/or hospitalization for diabetic ketoacidosis in the last 6 mo before the screening visit; or unstable proliferative diabetic retinopathy or any other rapidly progressive diabetic retinopathy or macular edema likely to require treatment during the study period.

Following a 6 mo treatment period, participants continued receiving previously assigned once-daily Glar-300 or Glar-100 plus OADs, in a 6 mo extension period. The authors evaluated the changes from BL to month 12 in HbA1c, laboratory-measured FPG, average preinjection SMPG, and average 7-point SMPG, as well as daily basal insulin dose, mean 7-point SMPG profiles at BL and month 12, hypoglycemic events, body weight, and AEs during the 12 mo period [55, 56].

### 5.7. Primary and Secondary Outcomes

The primary outcome in all EDITION studies was change in HbA1c from BL to 6 mo (and BL to month 12), and the main secondary efficacy endpoints were

- Percentage of participants with hypoglycemic events [and the annualized event rates for hypoglycemia (events per participant-year), by a study period] and the cumulative mean number of hypoglycemic events per participant, categorized by the American Diabetes Association (ADA) [during the day (daytime; 06:00–23:59 h), any time of the day or night (24 h), and during the night (termed nocturnal, 00:00–05:59 h)]. The EDITION trials comprised three follow-up periods for the evaluation of hypoglycemia: titration phase (BL to week 8), maintenance phase (week 9 to month 6; this follow-up period was established to avoid the possibility of any temporary disruptions in the risk of hypoglycemia that could arise when switching from Glar-100, usually to Glar-300), and throughout the follow-up (BL to month 6 and BL to month 12),
- Change in preinjection SMPG and change in variability of preinjection SMPG,
- Development of AIAs,
- Change in daily basal insulin dose (U and U/kg body weight) and change in total insulin dose (basal plus mealtime) and in the daily insulin dose (U and U/kg body weight),
- Change in FPG,
- Proportion of patients with rescue therapy during the main 6 mo on-treatment period for EDITION 2, EDITION 3, and EDITION JP2,
- Change in the treatment satisfaction score using the DTSQ. Furthermore, EDITION 3 and 4 also evaluated the EQ-5D, an instrument that complements other forms of quality of life measurements and facilitates the collection of a number of common data for reference purposes. This trial also assessed fear to develop hypoglycemia using the HSF-II score, which is a survey that evaluates different aspects associated with the fear to present hypoglycemia under various circumstances [57–60].

### 5.8. Methods

The EDITION studies tested the main hypothesis of noninferiority of Glar-300 versus Glar-100 in terms of HbA1c lowering as required by regulatory agencies to register a new insulin preparation. In addition, EDITION 4 assessed noninferiority of morning versus evening injection of Glar-300 or Glar-100. The sample sizes in the Japanese trials were determined to satisfy regulatory requirements to gain marketing authorization and as such were designed purely based on demonstrating noninferiority in HbA1c change. Noninferiority was assessed for the primary endpoint; the upper bound of the two-sided 95% confidence interval (CI) for the difference in the mean change in HbA1c from BL to endpoint between Glar-300 and Glar-100 was compared with the predefined noninferiority margin of 0.4% HbA1c. Noninferiority was considered demonstrated if the upper bound of the two-sided 95% CI of the difference between Glar-300 and Glar-100 in the mITT population was <0.4%. If noninferiority was demonstrated, superiority of Glar-300 over Glar-100 was tested; the superiority of Glar-300 over Glar-100 was demonstrated if the upper bound of the two-sided 95% CI for the difference in the mean change in HbA1c from BL to endpoint between Glar-300 and Glar-100 in mITT population was <0.

If the primary endpoint was met, then to control for type I error, a hierarchical step-down testing procedure was applied as follows (the test was stopped as soon as an endpoint was found that was not statistically significant at one-sided *α* = 0.025 level). The primary efficacy population used was the mITT population, defined in the statistical analysis plan for each study as “all randomized patients who receive at least 1 dose of the open-label investigational medical product and have both a BL assessment and at least 1 post-BL assessment of any primary or secondary efficacy variables, irrespective of compliance with the study protocol and procedures.” The remaining population that was required for the mixed model for product repeated measurements was used for the statistical analysis.

Safety endpoints were analyzed descriptively using the safety population (all randomized participants exposed to at least one dose of the study treatment). The primary efficacy endpoint (outcome) was analyzed using analysis of covariance, with the difference between treatment groups expressed as the least squares (LS) mean difference in HbA1c change, having 2-sided 95% CI. The nocturnal time period (from 23.00–7.00 h to 00.00–05.59 h) in all phase 3 studies was adjusted. Nocturnal hypoglycemia was defined by this time period whether the patient was awake or asleep. The reason for the time adjustment was to try to exclude confounding factors (i.e., exercise, food, and mealtime insulin).

In EDITION 2, 3, and JP2 trials, if FPG or HbA1c measurements were above the target values and no reasonable explanation existed for insufficient glucose control or if appropriate action failed to decrease FPG/HbA1c under the threshold values, intensification of the treatment was considered. The choice of the glucose-lowering treatment added to the basal insulin was based on the investigator's decision and local labeling documents [45, 47, 49, 51, 53, 55].

### 5.9. Results

In the EDITION 1 trial, the mean HbA1c decreased in the two treatment groups; at the end of the treatment, HbA1c was 7.25% (SD: 0.85) [(55.7 mmol/mol (9.3)] with Glar-300 versus 7.28% (0.92) [56.1 mmol/mol (10.1)] with Glar-100. The LS mean change was −0.83% (SE: 0.06) [−9.1 mmol/mol (0.7)] for both groups; the difference was −0.00% (95% CI: −0.11 to 0.11) and −0.00 mmol/mol (95% CI: −1.2 to 1.2).

Reductions in laboratory-measured FPG from BL were observed in both treatment groups [from 8.72 mmol/L (SD: 2.83) to 7.24 mmol/L (2.57), or 157 mg/dL to 130 mg/dL, with Glar-300 and 8.90 mmol/L (2.94) to 7.21 mmol/L (2.40), or 160 mg/dL to 129.8 mg/dL, with Glar-100]. The percentages of participants attaining target HbA1c levels < 7.0% (53 mmol/mol) were 39.6% for Glar-300 and 40.9% for Glar-100, and those attaining target FPG < 5.6 mmol/L (100 mg/dL) were 26.5% for Glar-300 and 23.2% for Glar-100. The reduction in preinjection SMPG (combination of predinner and postdinner measurements) from BL to month 6 was similar between treatments [LS mean change: −0.90 mmol/L (SE: 0.18) for Glar-300 and −0.84 mmol/L (0.18) for Glar-100]. There was also no between-treatment difference in the change of day-to-day variability of preinjection SMPG during treatment.

Daily basal insulin dosage increased from 0.67 (SD: 0.29) to 0.97 (0.37) U/kg/d (70 U/d to 103 U/d) at the end of the 6 mo treatment period with Glar-300 and from 0.67 (SD: 0.28) to 0.88 (0.32) U/kg/d (71 U/d to 94 U/d) with Glar-100. Mealtime insulin doses increased in the first 2 wk but were unchanged from BL and alike in the two groups thereafter [final 0.55 (SD: 0.35) U/kg/d]. The final total daily dosage was 1.53 (0.61) U/kg/d with Glar-300 and 1.43 (0.6) U/kg/d with Glar-100. At month 6, the doses (mean U/kg) were 103.3 for Glar-300 and 93.7 for Glar-100. And the unit difference in mean unit basal insulin (mean difference in U/kg; defined as the difference between mean basal doses of Glar-300 and mean basal doses of Glar-100) was 9.6 [additionally, the percentage of difference in basal insulin (U/kg) was +11.4%].

The total daily insulin (mean U/kg) was 163 in the Glar-300 randomized group versus 154.2 in the Glar-100 randomized group. The unit difference in total daily insulin (mean difference in U/kg) which was defined as the difference between meal total Glar-300 insulin and meal total Glar-100 insulin was 8.8, and the percentage of difference in total daily insulin (U/kg) was 7.7%. At month 6, there was an increase in body weight in both treatment groups, from a BL (mean ± SD) of 106.11 ± 21.43 to 107.04 ± 21.86 in the Glar-300 group and of 106.5 ± 19.94 to 107.4 ± 20.33 in the Glar-100 group. The change from BL to month 6 by the last observation carried forward was +0.93 in the Glar-300 group and +0.90 in the Glar-100 group.

Over the program extension period of the EDITION 1 trial, of the 807 participants randomized during the initial treatment phase (404 in the Glar-300 group and 403 in the Glar-100 group), 359 (89%) and 355 (88%), respectively, completed the follow-up. At month 12, the mean HbA1c with Glar-300 was 7.24% (SD: 0.93) and that with Glar-100 was 7.42% (0.94). The LS mean change from BL was −0.86% with Glar-300 and −0.69% with Glar-100 (the LS mean difference between reductions with Glar-300 and Glar-100 at month 12 was −0.17% (95% CI: −0.30 to −0.05; *P* = 0.007). A similar pattern in the change of laboratory-measured clinic-collected FPG at month 6 was observed at month 12; the mean change from BL was −1.6 mmol/L (−29.6 mg/dL) for Glar-300 and −1.4 mmol/L (−26 mg/dL) for Glar-100 [the LS mean difference between reductions with Glar-300 and Glar-100 was −0.34 mmol/L (95% CI: −0.69 to 0.01) or −6.1 mg/dL (95% CI: −12.5 to 0.2) (*P* = 0.058)].

Over 12 mo of treatment, the daily basal insulin dose in both treatment groups increased from a BL value of 0.67 U/kg; the increase in dose occurred predominantly during the first 12 wk. At month 12, the mean daily basal insulin dose was 1.03 U/kg (SD: 0.40) with Glar-300 and 0.90 U/kg (0.35) with Glar-100. The mean daily mealtime insulin dose at 12 mo was 0.55 U/kg (0.36) and 0.56 U/kg (0.38) with Glar-300 and Glar-100, respectively (the corresponding values for the total daily insulin dose were 1.58 U/kg (0.66) and 1.45 U/kg (0.62). Finally, the mean weight change from BL to the last on-treatment value was 1.2 kg (3.8) with Glar-300 and 1.4 kg (3.5) with Glar-100 [LS mean difference for Glar-300 versus Glar-100: −0.2 kg (95% CI: −0.7 to 0.3)] [45, 46].

In the EDITION 2 trial, the mean HbA1c at month 6 was 7.57% (59.2 mmol/mol) in the Glar-300 group and 7.56% (59.1 mmol/mol) in the Glar-100 group. The LS mean change was −0.57% (SE: 0.09) or −6.2 mmol/mol (1.0) for Glar-300 and −0.56% (0.09) or −6.1 mmol/mol (1.0) for Glar-100, with a mean difference of −0.01% (SE: 0.07; 95% CI: −0.14 to 0.12). Similar proportions of participants reached target HbA1c < 7.0% and ≤6.5% (30.6% and 14.5% with Glar-300 and 30.4% and 14.8% with Glar-100, resp.). Moreover, the proportions of participants attaining FPG ≤ 6.7 mmol/L (120 mg/dL) or <5.6 mmol/L (100 mg/dL) were 48.7 and 29.4% for Glar-300 versus 54.1 and 33.6% for Glar-100.

In both treatment groups, FPG declined mostly in the first 12 wk of therapy with a treatment mean difference of Glar-300 versus Glar-100 of 3.38 mg/dL (95% CI: −2.670 to 9.435). Despite the decrease in FPG in both groups, there was a larger adjusted decrease for Glar-100 (−21.9 mg/dL) compared to Glar-300 (−18.5 mg/dL). 24 h average SMPG results (from 8-point SMPG profiles) showed that there was a transient increase in SMPG values in the Glar-300 compared to the Glar-100 group at week 4. SMPG values decreased similarly between the two groups up to week 12, and at month 6, a similar average prebreakfast SMPG was reached in both groups [6.59 mmol/L (119 mg/dL) for Glar-300 and 6.28 mmol/L (113 mg/dL) for Glar-100].

The dose of insulin in the Glar-300 group at the endpoint (month 6) was 91 U (0.92 U/kg), an increase from 62.1 U (0.64 U/kg) at BL. Likewise, daily Glar-100 dose at the end of month 6 was 81.9 U (0.84 U/kg), an increase from 63.9 U (0.66 U/kg) at BL. The unit difference in mean unit basal insulin was 9.1, and the percentage of difference in basal insulin was +11.9%. Finally, the percentage of difference in total daily insulin was +11.9%. The mean ± SD increase in body weight from BL to month 6 was numerically lower in the Glar-300 group compared to the Glar-100 group (0.08 ± 3.45 kg versus 0.66 ± 3.01 kg, resp., *P* = 0.015). The percentage of patients who needed rescue therapy during the main 6 mo on-treatment period was 5.7% (23 patients) in the Glar-300 group and 4.9% (20 patients) in the Glar-100 group. The most frequent rescue therapy used was rapid-acting insulin analogues.

The efficacy and safety of Glar-300 versus Glar-100 after 1 year of treatment showed that the glycemic control achieved with Glar-300 and Glar-100 was similar. The LS mean difference in HbA1c between treatment groups at month 12 was 0.06% (95% CI: −0.22 to 0.10%). FPG was similarly improved in both groups; LS mean difference in change from BL in FPG between groups at month 12 was 0.2 mmol/L (95% CI: −0.2 to 0.6) or 3.6 mg/dL. The basal insulin dose was higher by an average 0.11 U/kg/d (approximately 12%) with Glar-300 than with Glar-100 at month 12. The overall weight gain at month 12 from BL was lower with Glar-300 versus Glar-100 [LS mean change of 0.42 kg (0.04 to 0.80) for Glar-300 and 1.14 kg (0.76 to 1.52) for Glar-100; LS mean difference of −0.72 kg (−1.25 to −0.18); *P* = 0.0091]. During the 12 mo on-treatment period, 33 participants (8.2%) in the Glar-300 group and 41 (10.1%) in the Glar-100 group received rescue therapy [47, 48].

In the EDITION 3 trial, the analysis showed a decrease in HbA1c in Glar-300 (mean ± SD) from a BL of 8.49 ± 1.04% to 7.08 ± 0.96% (a difference of −1.40 ± 1.10% from BL). In the Glar-100 group, the HbA1c (mean ± SD) decreased from a BL of 8.58 ± 1.07% to 7.05 ± 0.95% (a difference of −1.53 ± 1.19% from BL). The LS mean difference in change in HbA1c was 0.04% (95% CI: −0.09 to 0.17) or 0.4 mmol/mol (95% CI: −1.0 to 1.9). Although Glar-300 and Glar-100 had decreases in FPG throughout the 6 mo treatment period, the adjustment between group differences (+6.99 mg/dL, 95% CI: 1.8 to 12.2) showed that despite higher dosages of Glar-300, achieved FPG was higher than that of Glar-100. 24 h average SMPG results (from 8-point SMPG profiles) showed that there were higher SMPG values in the Glar-300 group compared to the Glar-100 group after week 2 until the end of the study; nevertheless, similar findings were seen in the average prebreakfast SMPG values during the 6 mo on-treatment period, although the prebreakfast SMPG decreased more gradually with Glar-300.

Throughout the study, the basal insulin dose increased in both groups, with the increase in the Glar-300 group being greater than that in the Glar-100 group at month 6 [Glar-300: 59.4 U (0.62 U/kg) and Glar-100: 52 U (0.53 U/kg)]. The Glar-300 group required 7.4 more units of insulin than the Glar-100 group [the doses (mean U) at month 6 were 59.4 for Glar-300 and 52 for Glar-100], and the percentage of difference in basal insulin was +14.8% (the percentage of difference in total daily insulin was +14.8%). The mean ± SD change in body weight from BL to month 6 was 0.50 ± 3.70 kg in the Glar-300 group versus 0.71 ± 3.61 kg in the Glar-100 group [LS mean increase of 0.49 kg (95% CI: 0.14 to 0.83) with Glar-300 versus LS mean increase of 0.71 kg (95% CI: 0.36 to 1.06) for Glar-100]. Finally, the percentage of patients in whom rescue therapy was initiated during the main 6 mo on-treatment period was 1.6% (7 patients) for Glar-300 and 3.5% (15 patients) for Glar-100; rescue treatment was initiated mostly after 90 d of study treatment.

Over the program extension period (at month 12) in the EDITION 3 trial, of the 878 participants randomized in the initial treatment phase (439 in the Glar-300 group and 439 in the Glar-100 group), 432 receiving Glar-300 and 430 receiving Glar-100 comprised the mITT population, and 337 participants in the Glar-300 group and 314 in the Glar-100 group completed the follow-up. At month 12, mean (SD) HbA1c was 7.13% (1.0) or 54.4 mmol/mol (10.9) with Glar-300 and 7.24% (0.97) or 55.6 mmol/mol (10.6) with Glar-100. The LS mean difference in HbA1c change from BL to month 12 for Glar-300 versus Glar-100 was −0.08% (95% CI: −0.23 to 0.07) or −0.9 mmol/mol (95% CI: −2.5 to 0.8). The percentage of participants reaching an HbA1c target of <7.0% without nocturnal (00:00–05:59 h) confirmed [≤3.9 mmol/L (70 mg/dL)] or severe hypoglycemia was 23% for Glar-300 and 19% for Glar-100 [responder ratio: 1.24 (95% CI: 0.96 to 1.61)]. For those without nocturnal documented symptomatic (≤70 mg/dL) hypoglycemia, the percentage of participants was 28% versus 24% (responder ratio: 1.19; 95% CI: 0.96 to 1.49).

The LS mean difference in FPG change for Glar-300 versus Glar-100 was 0.07 mmol/L (95% CI: −0.26 to 0.40) or 1.32 mg/dL (95% CI: −4.62 to 7.26). The mean (SD) prebreakfast SMPG levels were 6.19 mmol/L (1.21) or 111.5 mg/dL (21.8) for Glar-300 and 6.18 mmol/L (1.37) or 111.4 mg/dL (24.7) for Glar-100. Eight-point SMPG profiles decreased markedly for both Glar-300 and Glar-100 during the study, and at month 12, the plasma glucose profiles were similar in the two treatment groups. Daily basal insulin dose increased up to month 12 in both treatment groups, with the mean (SD) basal insulin dose at month 12 being 0.67 U/kg/d (0.33) for the Glar-300 group and 0.56 U/kg/d (0.27) for the Glar-100 group (20% higher with Glar-300; the 45% of the dose difference at month 12 was reached by week 12).

The mean (SD) change in body weight from BL to the last on-treatment value was 0.97 kg (4.32) for Glar-300 and 1.20 kg (4.16) for Glar-100. LS mean difference was −0.24 kg (95% CI: −0.81 to 0.33). The majority of discontinuations in the Glar-300 (57 participants) and Glar-100 (78 participants) groups were made at the participant's request. Perceived lack of efficacy accounted for the discontinuation of 3 participants (0.7%) in the Glar-300 group and 1 participant (0.1%) in the Glar-100 group. Rescue therapy was required by 15 (3.4%) and 26 (5.9%) participants in the Glar-300 and Glar-100 groups, respectively [49, 50].

In the EDITION 4 trial, the HbA1c in the Glar-300 group (mean ± SD) decreased from a BL of 8.13 ± 0.77% to 7.70 ± 0.99% (a difference of −0.42 ± 0.98% from BL). In the Glar-100 group, the HbA1c (mean ± SD) decreased from a BL of 8.12 ± 0.79% to 7.68 ± 0.80% (a difference of −0.44 ± 0.72% from BL). The between-drug group LS mean difference ± SE was 0.04 ± 0.072% (95% CI: −0.098% to 0.185%). The percentage of patients that achieved a level of HbA1c < 7.0% at month 6 was 16.8% for Glar-300 versus 15.0% for Glar-100. In the morning injection groups, the change from BL in HbA1c was −0.48% for Glar-300 and −0.41% for Glar-100; in the evening injection groups, the change from BL in HbA1c was −0.32% for Glar-300 and 0.48% for Glar-100.

24 h average SMPG results (from 8-point SMPG profiles) showed that there was a transient increase in SMPG values in the Glar-300 compared to the Glar-100 group at week 2; nevertheless, similar findings were seen in the average prebreakfast SMPG values during the 6 mo on-treatment period. Overall, it appears that SMPG values were higher for Glar-300 than for Glar-100 during the duration of the main 6 mo on-treatment period. Additionally, for the Glar-300 group, the 8-point SMPG profiles with morning and evening injection look similar, whereas for Glar-100, a difference appears in prebreakfast. Moreover, although BL levels of prebreakfast SMPG were somewhat higher on Glar-100, levels at 6 mo were similar, and the laboratory-measured clinic FPG decreased to 175.5 mg/dL (SD: 71.4) in the Glar-300 group and to 173.5 mg/dL (69.4) in the Glar-100 group.

At month 6, the mean total daily insulin dose (for basal insulin) was 40.5 U (0.47 U/kg) for the Glar-300 group and 34.1 U (0.40 U/kg) for the Glar-100 group (with a percentage of difference in basal insulin of +17.5%). The prandial dose was 28.7 (0.34 U/kg) for Glar-300 and 27.1 (0.33 U/kg) for Glar-100. In general, the total insulin doses were 69.6 (0.81 U/kg) for Glar-300 and 60.9 (0.73 U/kg) for Glar-100 (with a percentage of difference in total daily insulin of +11%). At month 6, the Glar-300 group required 6.4 more units of basal insulin and 1.6 more units of prandial insulin than the Glar-100 group; overall, the Glar-300 group required 8.7 more units of total insulin than the Glar-100 group.

For the Glar-300 group, mealtime insulin doses were relatively stable, but for Glar-100, there was some fall in the morning group and a rise in the evening group. Finally, after 6 mo of treatment, the Glar-300 overall group had a mean increase in body weight of +0.50 kg (SE: 3.3) versus +1.02 kg (3.2) in the Glar-100 overall group [with a difference of −0.6 kg (95% CI: −1.1 to −0.03; *P* = 0.037)]. At month 12, 219 participants (80%) in the Glar-300 group and 225 (82%) in the Glar-100 group completed the treatment period. The change in the HbA1c was similar from BL to month 12 in the Glar-300 and Glar-100 groups [−0.2% (SD: 0.06) for Glar-300 and −0.22 (0.06) for Glar-100]. The LS mean difference in change from BL was 0.02% (95% CI: −0.13 to 0.17) or 0.2 mmol/mol (95% CI: −1.5 to 1.9).

During the second 6 mo, mean HbA1c increased in both groups, remaining below BL and ending at 7.86% (SD: 1.03) or 62.4 mmol/mol (11.3) for Glar-300 and 7.86% (0.84) or 62.4 mmol/mol (9.2) for Glar-100. When comparing morning and evening injections, there was no difference in HbA1c change over 12 mo for Glar-100; however, the Glar-300 morning injection group ended at 7.76% (0.98) or 61.4 mmol/mol (10.7) and the Glar-300 evening injection group at 7.96% (1.07) or 63.5 mmol/mol (11.7) [LS mean difference in change from BL: −0.25% (95% CI: −0.47 to −0.04) or −2.7 mmol/mol (95% CI: −5.2 to −0.4)]. The LS mean difference in change from BL to month 12 in laboratory-measured clinic FPG was 0.18 mmol/L (95% CI: −0.55 to 0.90) or 3.2 mg/dL (95% CI: −10.0 to 16.3).

No effect of injection time was observed for the time course of laboratory-measured FPG, with comparable reductions by month 12 for evening versus morning groups. The LS mean difference (of average 24 h SMPG) for Glar-300 versus Glar-100 was 0.12 mmol/L (−0.34 to 0.58), and the change in the prebreakfast SMPG was −0.32 mmol/L (2.98) for Glar-300 and −0.76 mmol/L (2.71) for Glar-100. The mean (SD) daily basal insulin dose prior to the study was 0.38 (0.17) U/kg for Glar-300 and 0.37 (0.15) U/kg for Glar-100 and increased at month 12 for both groups (0.22 U/kg for Glar-300 and 0.18 U/kg for Glar-100).

The mean daily basal insulin doses at 12 mo for Glar-300 were 0.51 (0.23) U/kg for morning injections and 0.46 (0.21) U/kg for evening injections; for Glar-100, they were 0.45 (0.18) U/kg and 0.36 (0.17) U/kg. Total daily insulin dose at month 12 for morning injections was 15.6% higher for Glar-300 [0.87 (0.33) U/kg)] than for Glar-100 [0.75 (0.25) U/kg], and for evening injections, it was 16.8% higher [0.82 (0.36) versus 0.70 (0.28) U/kg]. Additionally, while in the morning injection group, the basal insulin was 58.2% of the total dose with Glar-300 and 59.5% with Glar-100, in the evening injection groups, these proportions were 56.1% and 51.7%. Finally, the mealtime total insulin dose remained stable in both groups over the study duration. Body weight increased in both treatment groups, but the statistically significant difference at 6 mo in favor of Glar-300 was lost at 12 mo [LS mean difference: −0.5 kg (95% CI: −1.1 to 0.1), *P* = 0.098] [51, 52].

In the EDITION JP1 trial, at month 6, the mean HbA1c level had decreased by 0.30% (SE: 0.06) with Glar-300 versus 0.43% (SE: 0.06) with Glar-100. The LS mean difference was 0.13% (95% CI: −0.03 to 0.29). The percentages of people receiving Glar-300 and Glar-100 who achieved HbA1c < 7.0% were 15.6% (19/122) and 20% (24/120), respectively. No between-treatment differences were observed in the change from BL to month 6 in FPG, with a LS mean difference between groups of 0.4 mmol/L (95% CI: −0.6 to 1.4 mmol/L) or 7.4 mg/dL (95% CI: −10.4 to 25.1).

At month 6, SMPG was consistently lower at all time points (demonstrated by average 8-point SMPG profiles) in the Glar-300 group compared with that at BL, whereas there was no consistent trend in the Glar-100 group. At month 6, mean predinner SMPG was significantly lower with Glar-300 [8.4 mmol/L (151.7 mg/dL)] versus Glar-100 [10.0 mmol/L (180.3 mg/dL)]; the LS mean difference was −1.6 mmol/L (95% CI: −2.8 to −0.3) or −28.1 mg/dL (95% CI: −50.1 to −6.1). Glycemic control was notably better with Glar-300 than Glar-100 from predinner to bedtime at month 6; the average preinjection SMPG was also significantly lower with Glar-300 [9.3 mmol/L (166.8 mg/dL)] versus Glar-100 [10.3 mmol/L (185.8 mg/dL)]; LS mean difference was −1.0 mmol/L (95% CI: −1.8 to −0.3) or −18.5 mg/dL (95% CI: −32.0 to −5.0).

The mean total insulin dose at month 6 was 0.79 (SD: 0.25) U/kg/d (basal insulin of 0.35 U/kg/d and mealtime insulin of 0.44 U/kg/d) for the Glar-300 group and 0.74 (0.22) U/kg/d (basal insulin of 0.29 U/kg/d and mealtime insulin of 0.45 U/kg/d) for the Glar-100 group. This corresponds to a mean total insulin dose of 50.7 (SD: 20.4) U/d (basal insulin of 23 U/d and mealtime insulin of 28 U/d) for the Glar-300 group and 46.0 (17.6) U/d (basal insulin of 18.2 U/d and mealtime insulin of 27.8 U/d) for the Glar-100 group. The mean body weight decreased by 0.1 kg (SE: 0.2) with Glar-300, whereas a weight gain of 0.4 kg (SE: 0.2) was observed with Glar-100 [LS mean difference: −0.6 kg (95% CI: −1.1 to 0.0; *P* = 0.0347)].

At month 12, all randomized participants were included in the mITT and safety populations, with 114 (93%) of the participants receiving Glar-300 and 114 (94%) of the participants receiving Glar-100 completing the 12 mo treatment period. The mean HbA1c was 7.9% (SD: 0.9) in the Glar-300 group and 7.8% (0.9) in the Glar-100 group. The mean change from BL to month 12 was −0.2% (SD: 0.8) with Glar-300 and −0.3% (0.7) with Glar-100. The mean laboratory-measured FPG at month 12 was 9.6 mmol/L (SD: 3.9) or 173 mg/dL (70.4) in the Glar-300 group and 9.8 mmol/L (4.4) or 175.9 mg/dL (80.1) in the Glar-100 group. The mean change from BL to month 12 was −0.8 mmol/L (4.8) or −14 mg/dL (86.5) with Glar-300 and −0.4 mmol/L (5.2) or −7.0 mg/dL (93.2) with Glar-100.

The mean average preinjection SMPG at month 12 was 9.6 mmol/L (2.8) or 173.7 mg/dL (51.2) with Glar-300 and 10.9 mmol/L (3.4) or 197 mg/dL (61.5) with Glar-100. The mean change in mean preinjection SMPG from BL to month 12 was −0.2 mmol/L (3.5) or −3.1 mg/dL (62.6) in the Glar-300 group compared with 1.1 mmol/L (3.9) or 18.9 mg/dL (70.5) in the Glar-100 group. The mean change in 24 h average plasma glucose (based on 7-point SMPG profiles) from BL to month 12 was −0.04 mmol/L (SD: 3.1) or −0.8 mg/dL (55.8) in the Glar-300 group and −0.12 mmol/L (3.2) or −2.1 mg/dL (58.3) in the Glar-100 group. The mean basal and mealtime insulin doses were 23.7 U/d (0.4 U/kg/d) and 28.5 U/d (0.5 U/kg/d), respectively, in the Glar-300 group and 17.6 U/d (0.3 U/kg/d) and 29.2 U/d (0.5 U/kg/d), respectively, in the Glar-100 group. With Glar-300, the majority of the dose increases occurred during the first 12 wk of treatment; the increase in mean mealtime insulin dose from BL to month 12 was 2 U/d (0.04 U/kg/d) in the Glar-300 group and 5 U/d (0.08 U/kg/d) in the Glar-100 group. The change in body weight between BL and month 12 was 0.06 kg (SE: 021) in the Glar-300 group and 0.41 kg (SE: 0.19) in the Glar-100 group [53, 54].

In the EDITION JP2 trial, Glar-300 met the primary endpoint of noninferiority for change in HbA1c over 6 mo compared with Glar-100 [LS mean difference: 0.10% (95% CI: −0.08 to 0.27) or 1.1 mmol/mol (95% CI: −0.9 to 3.0)]. Achievement of HbA1c < 7% was similar with both groups (25.0% in the Glar-300 group and 24.2% in the Glar-100 group). No between-treatment differences were observed in change from BL to month 6 in FPG [with a LS mean difference between groups of 0.04 mmol/L (95% CI: −0.4 to 0.49) or 0.8 mg/dL (95% CI: −7.3 to 8.8 mg/dL)]. Up to 34% (40/118) and 40% (48/119) of the participants achieved the FPG target of <5.6 mmol/L (<100 mg/dL) at month 6 with Glar-300 and Glar-100, respectively.

At month 6, the mean 8-point SMPG profiles showed a decrease in SMPG from BL at all time points for both treatments (but relatively small differences were observed between treatments). Between BL and month 6, average preinjection SMPG values increased in both treatment groups. The LS mean change from BL to month 6 was 0.7 mmol/L (SE: 0.29) or 13 mg/dL (SE: 5.2) with Glar-300 and 0.9 mmol/L (0.29) or 17 mg/dL (5.2) with Glar-100. Basal insulin doses increased from 16 U/d (0.23 U/kg/d) to 24 U/d (0.35 U/kg/d) with Glar-300 and from 16 U/d (0.24 U/kg/d) to 20 U/d (0.30 U/kg/d) with Glar-100 over 6 mo. At month 6, the mean daily basal insulin dose was 0.35 U/kg (SD: 0.17) with Glar-300 and 0.30 U/kg (0.14) with Glar-100 (representing an approximate 17% increase). There was a small reduction in body weight in the Glar-300 group [LS mean weight change was −0.6 kg (SE: 0.2) for Glar-300 compared with an increase of 0.4 kg (0.2) for Glar-100 (LS mean difference: −1.0 kg (95% CI: −1.5 to −0.5); *P* = 0.0003)].

At month 12, of the 241 randomized participants, 107/121 (88%) in the Glar-300 group and 115/120 (96%) in the Glar-100 group completed the 12 mo on-treatment period, and rescue medication was used by 7/121 (6%) participants in the Glar-300 group and 1/120 (1%) participants in the Glar-100 group. None of these participants permanently discontinued treatment during the 12 mo study. Reductions in HbA1c levels from BL were similar in the two treatment groups [LS mean difference: 0.0% (95% CI: −0.2 to 0.2)]. The mean (SD) HbA1c had decreased to 7.7% (0.9) or 60.8 mmol/mol (10.2) for the Glar-300 group and 7.7% (1.0) or 61 mmol/mol (10.6) for the Glar-100 group. The mean (SD) change in HbA1c from BL to month 12 was −0.3% (0.8) or −3.1 mmol/mol (9.2) with Glar-300 and −0.3% (0.8) or −3.6 mmol/mol (8.6) with Glar-100. The mean (SD) FPG also decreased from BL to month 12 to 7.0 mmol/L (2.8) or 126.5 mg/dL (50.0) in the Glar-300 group and 6.4 mmol/L (1.9) or 115.3 mg/dL (34.1) in the Glar-100 group; the mean (SD) change in FPG from BL was −0.7 mmol/L (3.1) or −12.1 mg/dL (56.6) with Glar-300 and −1.0 mmol/L (2.4) or −18.6 mg/dL (43.3) with Glar-100.

The mean (SD) change in average 7-point SMPG from BL to month 12 was −0.9 mmol/L (2.6) or −15.6 mg/dL (46.0) for Glar-300 and −0.4 mmol/L (2.5) or −6.8 mg/dL (44.4) for Glar-100. The mean basal insulin dose increased in both groups from BL to month 12, with most of the change occurring in the first 12 weeks of treatment. In the Glar-300 group, the mean (SD) change in daily basal insulin dose from BL to month 12 was 9.0 U (10.2) or 0.13 U/kg (0.13). In the Glar-100 group, the mean (SD) change in daily basal insulin dose was 4.8 U (6.9) or 0.06 U/kg (0.09). At month 12, the mean (SD) daily basal insulin dose was 25.1 U (15.0) or 0.36 U/kg (0.18) in the Glar-300 group and 20.6 U (11.6) or 0.30 U/kg (0.15) in the Glar-100 group. The mean (SD) change in weight was −0.7 kg (0.2) in the Glar-300 group and 0.5 kg (0.2) in the Glar-100 group (*P* = 0.0001) [55, 56].

The main glycemic responses of the participants in the EDITION clinical trial program are shown in Table 2.

### 5.10. Hypoglycemia

The risk of hypoglycemia was lower in subjects receiving Glar-300 treatment. For instance, in the EDITION 1 trial, the reduction in risk of nocturnal hypoglycemia (≤70 mg/dL) was 21% on average (and 16% at month 12). In terms of the definition of at-any-time hypoglycemia (≤70 mg/dL), the reduction was 14%, 7%, and 6% for the BL to week 8, BL to month 6, and BL to month 12 periods, respectively. At month 6, the proportion of participants with one or more confirmed (≤70 mg/dL) or severe nocturnal hypoglycemic events between the start of week 9 and month 6 was 36% for Glar-300 versus 46% for Glar-100; the analysis of this prespecified main measure of hypoglycemia demonstrated superiority of Glar-300 over Glar-100 [relative risk (RR): 0.79; 95% CI: 0.67 to 0.93; *P* = 0.0045]. The percentage of participants reporting severe hypoglycemia at any time of the day or night (24 h) was 5.0% for Glar-300 versus 5.7% for Glar-100 (RR: 0.87; 95% CI: 0.48–1.55).

With the exception of severe nocturnal hypoglycemic events, which were too few for meaningful analysis, the percentage of participants within each category of nocturnal events [any hypoglycemia: documented (≤70 and <54 mg/dL) symptomatic hypoglycemia and confirmed (≤70 and <54 mg/dL) or severe hypoglycemia] was lower for Glar-300 than for Glar-100 (RR: 0.72–0.78) throughout the course of treatment. The annualized rates for nocturnal events were lower with Glar-300 (RR: 0.60 to 0.78) across all categories of hypoglycemia other than severe events. The risks of at-any-time events, nocturnal and daytime together, were equivalent or lower for Glar-300.

Over 12 mo, the cumulative number of confirmed (≤70 mg/dL) or severe hypoglycemic events per participant increased at similar rates for Glar-300 and for Glar-100 throughout the period of observation; however, the event rates did not differ between treatments (22 versus 21 per participant-year; rate ratio: 1.06; 95% CI: 0.89 to 1.27). Annualized rates of nocturnal confirmed (≤70 mg/dL) or severe hypoglycemia did not differ significantly between treatments (2.88 versus 3.19 events per participant-year; rate ratio: 0.90; 95% CI: 0.70 to 1.16). When the more stringent hypoglycemia threshold (<54 mg/dL) was applied to the categories of documented symptomatic and confirmed or severe hypoglycemia, no significant between-treatment differences were seen. Severe hypoglycemia (any time of the day) was reported by 6.7% of Glar-300-treated and 7.5% of Glar-100-treated participants [45, 46].

Likewise, the EDITION 2 trial showed a similar pattern for the reduction in risk of nocturnal hypoglycemia (≤70 mg/dL), with 47% lower risk over the BL to week 8 period, 23% lower risk over the week 9 to month 6 period, and 29% lower risk over the BL to month 6 period. The risk reduction in the at-any-time risk of hypoglycemia in the EDITION 2 trial was statistically significant for the BL to week 8 and BL to month 6 periods (with an identified risk reduction of 22% and 10%, resp.). Furthermore, a risk reduction in the documented symptomatic at-any-time hypoglycemia < 54 mg/dL was identified (23% drop) over the BL to month 6 period. During the 6 mo of treatment, 123 participants (30.5%) in the Glar-300 group experienced 379 nocturnal hypoglycemic events and 169 participants (41.6%) in the Glar-100 group experienced 766 nocturnal hypoglycemic events; a significantly lower percentage of participants reported at least one nocturnal confirmed (≤70 mg/dL) or severe hypoglycemic event from week 9 to month 6 with Glar-300 (21.6%) versus Glar-100 (27.9%).

Analysis of this prespecified main secondary endpoint demonstrated superiority of Glar-300 over Glar-100 (RR: 0.77; 95% CI: 0.61 to 0.99; *P* = 0.038). The annualized rates of nocturnal confirmed (≤70 mg/dL) or severe hypoglycemia were 1.89 for Glar-300 and 3.68 for Glar-100 (RR: 0.52; 95% CI: 0.35 to 0.77; *P* = 0.0010). When assessed as a function of the value of HbA1c at the endpoint, the number of events per participant-year of nocturnal confirmed or severe hypoglycemia from week 9 to month 6 was lower in the Glar-300 group than in the Glar-100 group (*P* = 0.010). The annualized event rate for confirmed or severe hypoglycemia was statistically significantly lower for Glar-300 versus Glar-100 at 6 mo (14.01 versus 18.14, RR: 0.77; 95% CI: 0.63 to 0.96; *P* = 0.0175) and showed a more pronounced reduction during the first 8 wk (RR: 0.67; 95% CI: 0.51 to 0.86).

At month 12, there was a 37% relative reduction in annualized rate with Glar-300 compared with Glar-100 (1.74 versus 2.77, rate ratio: 0.63; 95% CI: 0.42 to 0.96; *P* = 0.0308). Considering all hypoglycemia, there were fewer nocturnal hypoglycemic events reported with Glar-300 (1.8 events per participant-year) versus Glar-100 (2.9 events per participant-year) (rate ratio: 0.61; 95% CI: 0.41 to 0.92). During the 12 mo study period, severe hypoglycemia at any time was reported by 7 (1.7%) participants (10 events) in the Glar-300 group and 6 (1.5%) participants (13 events) in the Glar-100 group, corresponding to a rate of 0.03 event per participant-year in both treatment groups. Three participants reported severe nocturnal hypoglycemic events [1 participant (0.2%) for Glar-300 and 2 participants (0.5%) for Glar-100] [47, 48].

On the other hand, a 24% reduction in the risk of nocturnal hypoglycemia (≤70 mg/dL) over the BL to month 6 period was observed in the EDITION 3 trial, with no benefits identified in the risk of at-any-time hypoglycemia. However, a reduced risk of documented symptomatic hypoglycemia < 54 mg/dL of 49% and 45% (nocturnal and at any time, resp.) was observed over the BL to month 6 period. Over the follow-up period for BL to month 12 of the EDITION 3 trial, only a benefit in a risk reduction in documented symptomatic hypoglycemia < 54 mg/dL (at any time) was documented.

The annualized event rates of nocturnal confirmed or severe hypoglycemia were similar in the two treatment groups during the 6 mo study period; the annualized event rate of hypoglycemia at any time was significantly lower with Glar-300 versus Glar-100 over 6 months (6.4 versus 8.5 events per participant-year, RR: 0.75; 95% CI: 0.57 to 0.99; *P* = 0.042) and showed a more pronounced reduction during the first 8 wk (4.5 versus 8.5 events per participant-year, RR: 0.61; 95% CI: 0.43 to 0.86). When considering both the percentage of participants experiencing and annualized rates of documented symptomatic (≤70 mg/dL) hypoglycemia at any time, results favored Glar-300 during all predefined study periods (RR: 0.42 to 0.85), with significant relative reductions in the annualized rate reported from BL to month 6 (RR: 0.62; 95% CI: 0.44 to 0.87) as well as during the first 8 wk (RR: 0.42; 95% CI: 0.26 to 0.67).

Significant reductions with Glar-300 versus Glar-100 were also apparent when considering the percentage of participants affected by events defined by the more stringent glycemic threshold. A 39% lower risk was observed for confirmed (<54 mg/dL) or severe hypoglycemia (RR: 0.61; 95% CI: 0.43 to 0.87) and a 45% lower risk for documented (<54 mg/dL) symptomatic hypoglycemia (RR: 0.55; 95% CI: 0.37 to 0.82) over the 6 mo period. Finally, severe hypoglycemia was infrequent, and events were too few for meaningful analysis. Only 4 participants (1%) in each treatment group reported severe hypoglycemia at any time.

At month 12, the annualized rates of nocturnal confirmed or severe hypoglycemic events were 1.33 events/participant-year for Glar-300 and 1.36 events/participant-year for Glar-100 (RR: 0.98; 95% CI: 0.69 to 1.40). The number of participants needed to be treated in order to prevent one participant from experiencing ≥1 confirmed or severe hypoglycemic event ≤ 70 mg/dL (at any time of the day) over 1 year was 21; the number of participants needed to be treated in order to prevent one participant from experiencing ≥1 confirmed or severe hypoglycemic event ≤ 70 mg/dL (nocturnal) over 1 year was 24. The annualized rates of hypoglycemic events were 7.14 events/participant-year for Glar-300 and 8.11 events/participant-year for Glar-100 (RR: 0.88; 95% CI: 0.70 to 1.11). Annualized rates of the events at any time of the day (documented symptomatic ≤ 70 mg/dL) showed a statistically significant 27% reduction in rate with Glar-300 versus Glar-100 (RR: 0.73; 95% CI: 0.54 to 0.99) [49, 50].

A significant reduction in the risk of nocturnal hypoglycemia (≤70 mg/dL) was documented in the EDITION 4 trial (−18%) over the BL to week 8 period, but no differences were found between Glar-300 and Glar-100 when the risk of hypoglycemia according to the time of administration of basal insulin was analyzed (morning or evening). The incidence rates were 78.4 (for Glar-300) and 72.5 events/person-year (for Glar-100) for hypoglycemia (at any time of the day) and 8.0 (for Glar-300) and 9.0 events/person-year (for Glar-100) for nocturnal hypoglycemia. In the preplanned analysis by the study period, the rate ratio for Glar-300 versus Glar-100 in the first 8 wk (using the definition of ≤70 mg/dL) was 0.69 (95% CI: 0.53 to 0.91).

Severe hypoglycemia was reported by 18 people (6.6%) in the Glar-300 group and by 26 (9.5%) in the Glar-100 group; of these, 6 (2.2%) and 7 (2.5%) people had nocturnal events. Annualized rates were 0.24 (for Glar-300) versus 0.34 (for Glar-100) events/person-year at any time of the day (and 0.08 versus 0.06 events/person-year during the night for Glar-300 versus Glar-100, resp.). When analyzed by morning or evening injection time, hypoglycemia in the Glar-300 group did not differ. Over 12 mo, 260 participants (in both treatment groups) had ≥1 confirmed (≤70 mg/dL) or severe hypoglycemic event [RR: 1.00 (95% CI: 0.96 to 1.05)]. A total of 199 participants (for the Glar-300 group) and 205 participants (for the Glar-100 group) had ≥1 nocturnal event [RR: 0.97 (95% CI: 0.88 to 1.08)].

Annualized rates of confirmed or severe hypoglycemia at any time of the day (24 h) were 75.9 and 68.8 events/person-year for Glar-300 and Glar-100, respectively [rate ratio: 1.11 (95% CI: 0.97 to 1.29)] and 8.1 and 8.6 events/person-year at night [rate ratio: 0.95 (95% CI: 0.75 to 1.20)]. The event rates for hypoglycemia over 12 mo were not statistically significant between Glar-300 and Glar-100 for all categories of definitions of hypoglycemia (at any time, nocturnal, threshold of ≤70 mg/dL or <54 mg/dL, confirmed or severe hypoglycemia, documented symptomatic hypoglycemia, and severe hypoglycemia) [51, 52].

Furthermore, in the EDITION JP1 trial, a reduction in the risk of nocturnal hypoglycemia (≤70 mg/dL) was identified to be 29% and 15% over the BL to week 8 and BL to month 6 periods, respectively; likewise, in terms of the risk of at-any-time hypoglycemia over the BL to week 8 period (9% reduction), in accordance with the definition of documented symptomatic hypoglycemia (nocturnal, <54 mg/dL), the EDITION JP1 trial showed a risk reduction of 36% over the BL to month 6 period and of 21% over the BL to month 12 period. The cumulative mean number of confirmed (≤70 mg/dL) or severe events per participant at any time (24 h) was lower with Glar-300 (rate ratio: 0.80; 95% CI: 0.65 to 0.98; *P* = 0.028). The cumulative mean number of confirmed (≤70 mg/dL) or severe events (events per participant-year) was lower with Glar-300 (rate ratio: 0.66; 95% CI: 0.48 to 0.92; *P* = 0.014). At month 12, the number of events (events per participant-year) was lower with Glar-300 for confirmed (≤70 mg/dL) or severe hypoglycemia (at any time) (RR: 0.86; 95% CI: 0.71 to 1.04) and for confirmed (<54 mg/dL) or severe hypoglycemia (nocturnal) (RR: 0.62; 95% CI: 0.39 to 0.97) [53, 54].

Finally, the EDITION JP2 trial showed a reduction in the risk of nocturnal hypoglycemia (≤70 mg/dL) over the week 9 to month 6, BL to month 6, and BL to month 12 periods (42%, 38%, and 27% drop, resp.), as well as in the risk of at-any-time hypoglycemia (≤70 mg/dL) over the BL to week 8 period (31% reduction). Confirmed (≤70 mg/dL) or severe hypoglycemia (nocturnal) over the 6 mo study period (annualized rates, events per participant-year) was lower with Glar-300 (rate ratio: 0.45; 95% CI: 0.21 to 0.96; *P* = 0.040) and for any time of the day (rate ratio: 0.64; 95% CI: 0.43 to 0.96; *P* = 0.030). At month 12, the annualized rate of confirmed (≤70 mg/dL) or severe hypoglycemia was lower with Glar-300 [RR: 0.41; 95% CI: 0.18 to 0.92 (nocturnal) and RR: 0.64; 95% CI: 0.44 to 0.94 (at any time)] [55, 56].

The risks of hypoglycemia for Glar-300 versus Glar-100 in participants with T2DM and T1DM in the program of the EDITION phase 3 clinical trials are shown in Table 3.

### 5.11. AEs

In the EDITION trials, AE was defined as any untoward medical occurrence in a clinical investigation in which a patient is administered a pharmaceutical product. The treatment-emergent (TE) AEs were defined as AEs that developed, worsened, or became serious during the main on-treatment period. If the treatment status for an AE was unclear due to missing or incomplete onset date, it was always considered treatment-emergent, unless otherwise shown by data. Serious (S) AEs were defined according to the internationally agreed-upon criteria outlined by the International Conference on Harmonization, in which a SAE is any untoward medical occurrence that at any dose results in death or is life-threatening.

The term “life-threatening” in the definition of “serious” refers to an event in which the patient was at risk of death at the time of the event; it does not refer to an event which hypothetically might have caused death if it were more severe. The event requires any of the three following criteria: inpatient hospitalization; prolongation of existing hospitalization, resulting in persistent or significant disability/incapacity; or cause of a congenital anomaly/birth defect. The term “severe” is used to describe the intensity (severity) of a specific event.

The rate and type of the following events were presented by the sponsor and were reviewed: deaths, SAEs, AE leading to dropout, systematically evaluated AEs [i.e., injection site, hypersensitivity reactions, cancers, cardiovascular events, hepatic events, and symptomatic overdose (accidental or intentional)], AEs in key demographic and BL subgroups, AEs related to pregnancy, changes from BL in laboratory variables, vital signs, body weight, electrocardiogram, and hypoglycemia [61–63].

The proportion of patients with TEAEs and other relevant results in the EDITION phase 3 clinical trial program are shown in Table 4.

### 5.12. Treatment Satisfaction (DTSQ Scores)

The treatment satisfaction scores were measured by the DTSQ [58].

In the EDITION 1 trial, the treatment satisfaction scores were similar between treatment groups and generally increased from BL to month 6, with a small between-treatment difference in favor of Glar-300 versus Glar-100. At month 6, more than half of the patients experienced a decrease from BL in the perception of hypoglycemia (in favor of Glar-300). At month 12, the improvements in the mean total DTSQ score from BL for Glar-300 and Glar-100 were similar. Improvements in perceived frequency of hypoglycemia and perceived convenience were also similar between groups [45, 46].

In the EDITION 2 trial, the LS mean change in the total treatment satisfaction score from BL to month 6 was similar in both groups (Glar-300 and Glar-100). The perceived frequency of hypoglycemia was also similar between groups. At month 12, the participants in both treatment groups reported high satisfaction throughout the study. DTSQ treatment satisfaction scores were similar in each group; the improvement in DTSQ scores observed at month 6 was maintained at month 12, and perceived frequency of hypoglycemia remained stable with either treatment [47, 48].

In the EDITION 3 trial, the DTSQ improved from BL to month 6 in both treatment groups. There was no change in the EQ-5D utility index score in either treatment group. Fear of hypoglycemia was low and decreased over the 6 mo study period in both treatment groups (with no significant differences between the two treated groups). At month 12, the DTSQ results were high in both treatment groups (without significant differences between both groups); the EQ-5D utility index score remained stable in both treatment groups throughout the 12 mo study, and the fear of hypoglycemia was very low at BL and decreased further to month 12 in both treatment groups. The mean (SD) total HFS-II score decreased from 0.52 (0.63) to 0.42 (0.47) for Glar-300 and from 0.61 (0.68) to 0.47 (0.53) for Glar-100 [49, 50].

In the EDITION 4 trial, the LS mean change in the total treatment satisfaction score to month 6 and month 12 was similar in both treatment groups and no notable differences were seen for morning versus evening injection in either treatment group. There were no intergroup differences when evaluating the EQ-5D utility index score and the HFS-II score [51, 52].

### 5.13. Immunogenicity

The AIA assessments were performed at a centralized laboratory using a validated AIA binding assay methodology. The sponsor's definitions of AIA status and titer categories are as follows: AIA status (positive, negative) (a patient is defined as AIA-positive if the patient is positive at any time during the main 6 mo on-treatment period) and AIA titer category (low, high) (AIA titer categories are defined for an AIA-positive patient using the maximal titer value over the main 6 mo on-treatment period). Categories are based on actual data and are defined as low if the maximal titer is <64 and as high if the maximal titer is ≥64.

The pooled AIA data for studies in patients with T2DM (EDITION 2 and EDITION 3) showed that at BL 41.6% in the Glar-300 group and 37.7% in the Glar-100 group were positive for AIAs. Including BL, the percentage of AIA-positive patients ranged from 37 to 44%, independent of the treatment group during all testing intervals. The percentage of patients showing a conversion of the antibody status from negative at BL to positive slightly increased from 10% at week 4 to about 20% in both groups at month 6. The percentage of patients who converted from BL positive to negative AIAs was about 20%. A number of patients with positive AIA titers in the Glar-300 (*n* = 16) versus Glar-100 (*n* = 17) groups experienced at least one severe hypoglycemia event. When analyzed by AIA titers, there were equal numbers of patients (2 patients) in each treatment group, with high AIA titers and who experienced at least one severe hypoglycemia event. Neither of these studies included insulin-naïve patients [47, 49].

In the EDITION 4 trial (patients with T1DM), at BL, a similar percentage of Glar-300 (169/274, 61.7%) and Glar-100 patients (147/275, 53.6%) was positive for AIAs. Throughout the main 6 mo on-treatment period, the percentage of AIA-positive patients slightly increased with increasing exposure; up to 78% (approximately) of patients in either treatment group had a positive AIA status during the 6 mo on-treatment period. Half as many patients in the Glar-300 group, AIA-positive patients experienced severe hypoglycemia compared to Glar-100 patients [11 (5.3%) versus 22 (10.4%)]. When analyzed by titers, there were more patients with high AIA titers in the Glar-300 group (3 patients) versus the Glar-100 group (1 patient), who experienced severe hypoglycemia. Over the 12 mo period, the percentage of participants who were positive at any time for AIAs was 89% for Glar-300 and 87% for Glar-100. The percentage of participants who were negative for AIAs at BL but positive later was 74% for both groups [51].

### 5.14. Substudies, Post Hoc Analysis, and Meta-Analysis of the EDITION Trials

Several substudies, post hoc analyses, and meta-analyses have been done based on the results of the EDITION trials. Such analyses have focused on various aspects, including the following: efficacy and safety of flexible versus fixed-dosing intervals of Glar-300 in T2DM; the effect of switching from twice-daily basal insulin to once-daily Glar-300 or Glar-100 in T2DM; evaluation of the nocturnal hypoglycemia risk for Glar-300 versus Glar-100, using different definitions of nocturnal hypoglycemia windows; evaluation of the impact of age of diabetes onset on glycemic control, as well as on hypoglycemia in T2DM treated with Glar-300 or Glar-100; efficacy and safety of Glar-300 using flexible-dosing or fixed-dosing intervals; evaluation of the effects of Glar-300 versus Glar-100 in different subgroups defined by age, BMI, and diabetes duration; the relationship between hypoglycemia and HbA1c in T2DM, comparing Glar-300 and Glar-100; the glycemic control and hypoglycemia benefits with Glar-300 in T2DM and mild-to-moderate renal impairment; and comparison of the efficacy and safety of Glar-300 and Glar-100 in older people (aged ≥ 65 years) with T2DM.

A patient-level meta-analysis of the EDITION 1, 2, and 3 trials compared the efficacy and safety of Glar-300 and Glar-100 in people with T2DM as related to basal and mealtime insulin, basal insulin and oral antihyperglycemic drugs, or no prior insulin, respectively. Of the 2496 participants included in the pooled analysis of the EDITION 1, 2, and 3 trials, 1247 were randomized to the Glar-300 group and 1249 to the Glar-100 group. The mITT population included 1239 participants with Glar-300 and 1235 participants with Glar-100; in the pooled dataset of all three studies, the LS mean change in HbA1c from BL to month 6 was −1.02% (SE: 0.03) for Glar-300 and −1.02% (0.03) for Glar-100 [LS mean difference: 0.00% (95% CI: −0.08 to 0.07%)].

The proportion of participants who reached target HbA1c < 7.0% after 6 mo of treatment was 449 participants (36.2%) for the Glar-300 group and 438 participants (35.5%) for the Glar-100 group. Laboratory-measured FPG decreased in both groups, with LS mean change at month 6 being −2.04 mmol/L (SE: 0.07) for Glar-300 and −2.26 mmol/L (0.07) for Glar-100 [LS mean difference: 0.21 mmol/L (95% CI: 0.03–0.40)]. Average preinjection SMPG also decreased in both treatment groups, and the reductions from BL to month 6 were similar for Glar-300 and Glar-100 [LS mean change: −1.43 mmol/L (SE: 0.08) and −1.34 (0.08); LS mean difference: −0.09 mmol/L (95% CI: −0.31 to 0.14)]. There was also no between-treatment difference in the variability of preinjection SMPG at month 6, with the LS mean at month 6 being 20% (SE: 0.32) for Glar-300 and 20% (0.33) for Glar-100 [LS mean difference: 0.02% (95% CI: −0.89 to 0.93)].

The annualized rate (events per participant-year) of confirmed or severe hypoglycemia at any time of the day over the 6 mo study period was 15.22 for Glar-300 and 17.73 for Glar-100 (rate ratio: 0.86; 95% CI: 0.77–0.97; *P* = 0.0116). The cumulative mean number of nocturnal confirmed or severe hypoglycemic events was lower with Glar-300 than with Glar-100. The annualized rates of nocturnal events over the 6 mo study period were 2.1 for the Glar-300 group and 3.06 for the Glar-100 group (rate ratio: 0.69; 95% CI: 0.57–0.84; *P* = 0.0002). A lower rate of hypoglycemia was shown during the night and beyond the predefined nocturnal period with Glar-300 compared with Glar-100.

Events were most frequently reported between 06:00 and 14:00 h [4777 (8.14 events per participant-year) in the Glar-300 group and 5925 (10.13 events per participant-year) in the Glar-100 group]. The number of participants who would need to be treated with Glar-300 in order to prevent 1 participant from having a confirmed or severe hypoglycemic event compared with Glar-100 was 16. For severe hypoglycemia, in the pooled analysis of all three studies, the number of participants with ≥1 event at any time of the day was 28 (2.3%) with Glar-300 and 33 (2.6%) with Glar-100 (RR: 0.85; 95% CI: 0.52 to 1.39). There was 0.11 event per participant-year in both groups (RR: 0.98; 95% CI: 0.51 to 1.86). Moreover, the mean basal insulin dose at month 6 was 0.85 U/kg/d (SD: 0.36) for Glar-300 and 0.76 U/kg/d (0.32) for Glar-100.

In relation to body weight, there was a slight weight gain with Glar-300 and Glar-100 [LS mean change: 0.51 kg (SE: 0.10) and 0.79 kg (0.10), resp.] in the pooled analysis of EDITION 1, 2, and 3 trials, but with less weight gain in the Glar-300-treated participants [LS mean difference: −0.28 kg (95% CI: −0.55 to −0.01); *P* = 0.039]. TEAEs were reported by 712 (57.3%) participants in the Glar-300 group and 669 (53.7%) participants in the Glar-100 group. Injection site reactions were reported for 30 (2.4%) participants in the Glar-300 group and 39 (3.1%) participants in the Glar-100 group. Serious TEAEs were reported by 65 (5.2%) and 62 (5.0%) participants in the Glar-300 and Glar-100 groups, respectively. Overall, 17 (1.4%) and 16 participants (1.3%) discontinued treatment because of a TEAE in the Glar-300 and Glar-100 groups, respectively, while 4 participants (0.3%) in the Glar-300 group and 3 (0.2%) in the Glar-100 group had TEAEs leading to death. AIA findings were similar between the treatment groups and across the three studies [64].

A substudy evaluated the efficacy and safety of Glar-300 when there is greater variability in the timing of injections, and two predefined 3-month substudies were embedded within EDITION 1 and EDITION 2, following the main 6 mo treatment period. In addition, 3-month substudies were conducted to examine the efficacy and safety of Glar-300 using flexible-dosing (24 up to 3 h) or fixed-dosing (24 h) intervals. Immediately after the main 6 mo treatment period of the EDITION 1 and EDITION 2 trials, eligible participants previously using Glar-300 were randomized (1 : 1) to continue with fixed dosing or to start using a flexible-dosing regimen.

Glar-300 was administered each evening, seeking a fasting self-measured plasma glucose of 80–100 mg/dL (4.4–5.6 mmol/L) prior to breakfast; the individuals in the flexible-dosing groups were instructed to inject insulin doses within 3 h earlier or later than their injection reference time (24 up to 3 h) and at the maximum interval (i.e., 3 h earlier or later than the reference injection time in the evening) on at least 2 d in each week. The individuals in the fixed-dosing groups were instructed to continue with the injection of basal insulin doses at their reference injection time each evening, with 24 h between injections.

The primary efficacy endpoint was the change in HbA1c from substudy BL to the end of the 3-month substudy. Secondary endpoints included the change in laboratory-measured (clinic-collected) FPG and daily basal insulin doses and hypoglycemia occurring between the substudy BL and the end of the substudy. In total, 109 patients from the EDITION 1 trial and 89 from the EDITION 2 trial were enrolled in the substudies. These 198 participants were randomized to either the flexible-dosing (*n* = 101) or fixed-dosing (*n* = 97) regimens. The safety population comprised 100 and 96 participants for the respective regimens, and the mITT population included 99 and 95 participants, respectively.

In the pooled analysis, 64% of the participants in the fixed-dosing group maintained all observed intervals in the 23–25 h range, compared with 15% of those in the flexible-dosing group. Of all intervals observed in the fixed-dosing group, 88% were in the 23–25 h range, whereas in comparison, 59% of intervals in the flexible-dosing group were in the 23–25 h range. Between-injection intervals outside the 21–27 h range were uncommon for the fixed-dosing group (2%), but 16% of intervals in the flexible-dosing group were outside this wider range. Within the individual substudies, the pattern of injection intervals was similar to that observed in the pooled data.

In the pooled analysis, HbA1c for both groups was similar to that observed at the substudy BL. The LS mean difference between regimens in the mean change in HbA1c was 0.05% (95% CI: −0.13 to 0.23). The laboratory-measured (clinic-collected) FPG levels were also similar at substudy BL and after 3 months for both treatment groups. The LS mean difference between the flexible- and fixed-dosing regimens in mean FPG change was 2.7 mg/dL (95% CI: −9.0 to 14.4) or 0.15 mmol/L (−0.50 to 0.80). The change in mean daily basal insulin dose was identical, with the LS mean difference being 0.00 U/kg (95% CI: −0.02 to 0.03) between the treatment groups.

The percentage of patients experiencing one or more nocturnal confirmed or severe hypoglycemic events was similar in the pooled data analysis (at 21% and 23% in the flexible-dosing and fixed-dosing groups, resp.), and the annualized rates of hypoglycemia compared using any definition, at any time (24 h) and during the night, were similar with the flexible-dosing and fixed-dosing regimens. Finally, the number of patients experiencing any TEAEs in the pooled data analysis was similar in both the flexible-dosing and fixed-dosing groups [24 (24%) and 26 (27%), resp.]. A low number of patients experienced serious AEs in the flexible- and fixed-dosing groups [6 (6%) and 5 (5%), resp.] [65].

A post hoc analysis of the EDITION 1 and EDITION 2 trials evaluated the effect of switching from twice-daily basal insulin to once-daily Glar-300 or Glar-100 on people with T2DM. The endpoints of post hoc analysis were change from BL to month 6 in HbA1c, change in basal insulin dose, percentage of participants with ≥1 confirmed (≤70 mg/dL) or severe hypoglycemic event, and change in body weight. At randomization, 16.9% and 20% of patients were receiving twice-daily basal insulin in the EDITION 1 trial and the EDITION 2 trial, respectively. Daily basal insulin dose in patients switching from twice-daily to once-daily insulin increased over 6 mo in both treatment groups, with a slightly higher dose for Glar-300 compared with Glar-100 [the change from BL to month 6 was 0.28 U/kg/d (SD: 0.21) versus 0.21 U/kg/d (0.22) for Glar-300 and Glar-100, respectively (in EDITION 1), and 0.36 U/kg/d (0.26) versus 0.24 U/kg/d (0.21) for Glar-300 and Glar-100, respectively (in EDITION 2)].

The switching from twice-daily basal insulin to once-daily Glar-300 or Glar-100 led to reductions in HbA1c, with comparable decreases in both treatment groups at month 6. The LS mean difference in change from BL in the HbA1c over 6 mo of treatment between prior twice-daily groups was −0.01% (95% CI: −0.27 to 0.24) and 0.16% (95% CI: −0.25 to 0.57) for the EDITION 1 and EDITION 2 trials, respectively. In both the EDITION 1 trial and the EDITION 2 trial, a lower risk of nocturnal confirmed or severe hypoglycemia was observed with once-daily Glar-300 versus once-daily Glar-100 in participants switching from twice-daily basal insulin. Similarly, a lower risk of confirmed or severe hypoglycemia at any time was also seen for Glar-300 versus Glar-100 in the EDITION 2 trial, regardless of the plasma glucose threshold.

Moreover, in the EDITION 1 trial, weight gain was comparable between treatment groups for participants who switched from twice-daily basal insulin [mean change from BL to month 6: 1.39 kg (SD: 3.50) for Glar-300 and 1.32 kg (3.13) for Glar-100]. In the EDITION 2 trial, a decrease in body weight was seen in participants treated with Glar-300 who switched from twice-daily basal insulin [mean change from BL to month 6: −0.71 kg (SD: 5.11)], while a slight increase was seen in the Glar-100 group [mean change: 0.58 kg (2.59)] [66].

A patient-level meta-analysis of people with T2DM (from the EDITION 2, 3, and JP2 trials) assessed the risk of nocturnal hypoglycemia for Glar-300 versus Glar-100 using four different windows to define nocturnal hypoglycemia. The prespecified hypoglycemia endpoints were the same for each study and were based on ADA definitions; confirmed or severe hypoglycemia was defined as any event that was documented symptomatic or asymptomatic with a plasma glucose measurement of ≤70 mg/dL or <54 mg/dL or severe. The events were reported as a pattern of hypoglycemia by the time of the day, percentage of participants with ≥1 event, and annualized rates (events per participant-year) during the main 6 mo treatment period. Hypoglycemia was assessed by a study and in a patient-level meta-analysis.

The windows used for evaluation of nocturnal hypoglycemia were per protocol, with events between 00:00 h and 05:59 h classified as nocturnal (predefined window); in this post hoc analysis, the predefined nocturnal interval was expanded by 2 h either in the late evening (22:00–05:59 h) or in the early morning (00:00–07:59 h). An additional window was defined using a fixed start time (22:00 h) and an end time that varied by a participant (based on each individual's recorded time of prebreakfast SMPG); finally, the percentage of participants with ≥1 hypoglycemic event and the rates of hypoglycemia per participant-year were estimated. In the patient-level meta-analysis, the median times of prebreakfast SMPG and basal insulin injection were 07:30 h (interquartile range [IQR]: 06:55–08:16) and 21:17 h (IQR: 20:00–22:05), respectively. At every time point, fewer participants reported confirmed or severe hypoglycemia for Glar-300 than for Glar-100. The events were reported most frequently between 06:00 h and 08:00 h; these events were only captured by windows extending beyond the predefined (00:00–05:59 h) window. The risk of ≥1 confirmed or severe event was consistently lower for Glar-300 than for Glar-100 using the predefined and the extended windows (the risk was 29% lower using the predefined window and 21-22% lower using the extended windows). A similar pattern of lower risk for Glar-300 versus Glar-100 was seen with other hypoglycemia definitions. For the annualized rates of nocturnal hypoglycemia for confirmed or severe hypoglycemia, rates were 41% lower using the predefined window and 29–34% lower using the extended windows [67].

In a post hoc patient-level meta-analysis of participants with T2DM treated with Glar-300 or Glar-100 for 6 mo (in the EDITION 1, 2, and 3 trials), the impact of age of diabetes onset on glycemic control and hypoglycemia was assessed. The subgroups were classified according to age of diabetes onset (<40 years, 40–50 years, and <50 years). The outcomes were as follows: change from BL to month 6 in HbA1c, basal insulin dose and body weight, percentage of participants with ≥1 confirmed or severe event (ADA categories), and events per participant-year (nocturnal and at any time).

The HbA1c reduction at month 6 was comparable between the Glar-300 and Glar-100 treatment groups, regardless of age of diabetes onset (no evidence of heterogeneity of treatment effect across subgroups, *P* = 0.56). In relation to participants with ≥1 hypoglycemic event, the lower risk of nocturnal confirmed or severe hypoglycemia with Glar-300 versus Glar-100 was not affected by age of diabetes onset (no evidence of heterogeneity of treatment effect across subgroups, *P* = 0.17). The benefit of Glar-300 in terms of lower risk of confirmed or severe hypoglycemia at any time was consistently seen, regardless of age of diabetes onset (no evidence of heterogeneity of treatment effect across subgroups, *P* = 0.31).

The hypoglycemia benefit of Glar-300 versus Glar-100 at night and at any time of the day was also seen when using the stricter hypoglycemic threshold of <54 mg/dL; the severe hypoglycemia at any time was infrequent (≤3.6% in all subgroups over 6 mo) and no evidence of heterogeneity of treatment effect was observed (*P* = 0.82). The results of hypoglycemic events per participant-year were consistent with the percentage of participants with ≥1 event (no evidence of heterogeneity of treatment effect was observed, *P* > 0.05). Moreover, the change in body weight over 6 mo was not affected by age of diabetes onset (no evidence of heterogeneity of treatment effect across subgroups, *P* = 0.57) [68].

A post hoc patient-level meta-analysis of the EDITION 1, 2, and 3 trials evaluated the effects of Glar-300 versus Glar-100 on HbA1c reduction and hypoglycemia in different subgroups [age (<65 and ≥65 years), BMI (<30 and ≥30 kg/m^2^), and diabetes duration (<10 and ≥10 years)] in T2DM (*n* = 2474). The LS mean HbA1c reduction over 6 mo of treatment (mITT population) was in overall −1.02% (SD: 0.03) for the Glar-300 group (*n* = 1239) and −1.02% (0.03) for the Glar-100 group [LS mean difference: −0.00% (95% CI: −0.08 to 0.07)]. HbA1c reduction remained comparable between the Glar-300 and Glar-100 arms, regardless of age, BMI, or disease duration [no evidence of heterogeneity of treatment effect across subgroups; *P* = 0.954 for age (<65 and ≥65 years), *P* = 0.665 for BMI (<30 and ≥30 kg/m^2^), and *P* = 0.067 for diabetes duration (<10 and ≥10 year)].

Hypoglycemia at any time of the day (24 h) in overall was 65.5% for the Glar-300 group and 72% for the Glar-100 group (RR: 0.91; 95% CI: 0.87 to 0.96). In patients < 65 years, the rate was 63.4% for Glar-300 and 70% for Glar-100 (RR: 0.9; 95% CI: 0.85 to 0.96), and in patients ≥ 65 years, it was 71.6% for the Glar-300 group and 77.4% for the Glar-100 group (RR: 0.93; 95% CI: 0.85 to 1.01). For patients with BMI < 30 kg/m^2^, the rate was 66.7% for the Glar-300 group and 74.7% for the Glar-100 group (RR: 0.89; 95% CI: 0.81 to 0.98); for patients with BMI ≥ 30 kg/m^2^, the rate was 65.1% for the Glar-300 group and 71.2% for the Glar-100 group (RR: 0.91; 95% CI: 0.86 to 0.96). For diabetes duration < 10 years, the rate was 55% for the Glar-300 group and 56.6% for the Glar-100 group (RR: 0.96; 95% CI: 0.86 to 1.07); for diabetes duration ≥ 10 years, the rate was 72.2% for the Glar-300 group and 82.5% for the Glar-100 group (RR: 0.88; 95% CI: 0.84 to 0.93).

The heterogeneity of treatment effect across subgroups (*P* value) for hypoglycemia at any time of the day (24 h) was 0.927, 0.654, and 0.006 for age, BMI, and diabetes duration, respectively. For nocturnal hypoglycemia in overall, the rates were 30% and 39.8% for the Glar-300 and Glar-100 groups, respectively (RR: 0.75; 95% CI: 0.68 to 0.83). For age < 65 years, the rates were 29.3% and 38% for the Glar-300 and Glar-100 groups, respectively (RR: 0.77; 95% CI: 0.68 to 0.87); for age ≥ 65 years, they were 31.8% and 44.9% for the Glar-300 and Glar-100 groups, respectively (RR: 0.70; 95% CI: 0.57 to 0.85). For patients with BMI < 30 kg/m^2^, the rates were 29.7% and 40.4% for the Glar-300 and Glar-100 groups, respectively (RR: 0.73; 95% CI: 0.59 to 0.91); for patients with BMI ≥ 30 kg/m^2^, they were 30.1% and 39.6% for the Glar-300 and Glar-100 groups, respectively (RR: 0.75; 95% CI: 0.67 to 0.85). For patients with diabetes duration < 10 years, the rates were 21.8% and 26.3% for Glar-300 and Glar-100, respectively (RR: 0.81; 95% CI: 0.65 to 1.01)]; for patients with diabetes duration ≥ 10 years, they were 34.9% and 48.9% for Glar-300 and Glar-100 groups, respectively (RR: 0.73; 95% CI: 0.64 to 0.82). The heterogeneity of treatment effect across subgroups (*P* values) for nocturnal hypoglycemia was 0.366, 0.774, and 0.109 for age, BMI, and diabetes duration, respectively [69].

On the other hand, a meta-analysis was performed on patient-level data, and analyses were also performed on data from individual studies. The estimated annualized rates (number of events per participant-year) as a function of HbA1c at month 6 were derived using a negative binomial model with the total number of events that occurred from BL to month 6 as the response variable, treatment and HbA1c at month 6 as covariates, and log-transformed period duration (from BL to month 6) as an offset variable. The objective was to explore the relationship between hypoglycemia over 6 mo and HbA1c at month 6 in T2DM comparing Glar-300 and Glar-100, using the data from the EDITION 1, 2, and 3 trials. The number of participants was 1247 and 1249 for Glar-300 and Glar-100, respectively (patient-level meta-analysis); in total, 1055 and 1048 participants with available hypoglycemia and HbA1c data from the Glar-300 and Glar-100 groups, respectively, were included in the meta-analysis.

The annualized rates of confirmed or severe hypoglycemia at night and hypoglycemia at any time of the day were lower with Glar-300 versus Glar-100, regardless of HbA1c values at month 6. Adding a treatment-by-HbA1c interaction term to the model did not significantly improve the goodness of fit (interaction *P* values were 0.829 and 0.937 for nocturnal hypoglycemia and at-any-time hypoglycemia, resp.). The authors concluded that, in the EDITION 1 trial, rates of hypoglycemia were likely confounded by the fact that patients were taking mealtime insulin in addition to basal insulin and that participants in the EDITION 3 trial were insulin naïve prior to the study and experienced fewer hypoglycemic events than those in the EDITION 1 and 2 trials (which possibly affected the ability to detect differences in the rates of hypoglycemia between the Glar-300 and Glar-100 groups in the EDITION 3 trial) [70].

In a post hoc patient-level meta-analysis of data from the EDITION 1, 2, and 3 trials, the effects of Glar-300 versus Glar-100 on HbA1c reduction and hypoglycemia were assessed in renal function subgroups [BL estimated glomerular filtration rate (eGFR) ≥30 to <60, ≥60 to <90, and ≥90 (mL/min/1.73 m^2^)]. In overall, the number of randomized participants was 1240 and 1236 for Glar-300 and Glar-100 groups, respectively. In the Glar-300 and Glar-100 groups, respectively, there were 201 (16%) and 200 (16%) participants with eGFR ≥ 30 to <60, 703 (57%) and 687 (56%) participants with eGFR ≥ 60 to <90, and 336 (27%) and 349 (28%) with eGFR ≥ 90.

HbA1c change from BL to month 6 (mITT population) in overall (LS mean) was −1.02% (SE: 0.03) for the Glar-300 group and −1.01% (0.03) for the Glar-100 group; the LS mean difference was −0.01% (95% CI: −0.08 to 0.06). In participants with eGFR ≥ 30 to <60, the change was −1.0% (SE: 0.07) for the Glar-300 group and −1.10% (0.07) for the Glar-100 group, with a LS mean difference of −0.1% (95% CI: −0.08 to 0.28). In participants with eGFR ≥ 60 to <90, the change was −1.06% (SE: 0.04) for the Glar-300 group and −1.04% (0.04) for the Glar-100 group; the LS mean difference was −0.03% (95% CI: −0.12 to 0.07). In participants with eGFR ≥ 90, the change was −0.95% (SE: 0.05) for the Glar-300 group and −0.92% (0.05) for the Glar-100 group; the LS mean difference was −0.03% (95% CI: −0.17 to 0.11). No evidence of heterogeneity of treatment effect was demonstrated across the subgroups (*P* = 0.46).

The risk of confirmed or severe hypoglycemia was significantly lower for nocturnal events and comparable or lower for at-any-time events for Glar-300 versus Glar-100 across subgroups. Renal function did not affect the lower rate of nocturnal or at-any-time hypoglycemia. There was no evidence of heterogeneity of treatment effect across subgroups (*P* = 0.73 and *P* = 0.27 for the rate ratio of nocturnal or at-any-time hypoglycemia, resp.) [71].

In other post hoc patient-level meta-analysis of the EDITION 1, 2, and 3 trials, the efficacy and safety of Glar-300 and Glar-100 were compared in older people (aged ≥ 65 years) with T2DM, over 12 mo of treatment. The mean HbA1c at month 12 was 7.23% (SD: 0.85) in the Glar-300 group and 7.29% (0.92) in the Glar-100 group. HbA1c reduction over 12 mo was comparable between treatment groups [LS mean difference between groups in change from BL to 1 year was −0.07% (95% CI: −0.21 to 0.07)]. The ADA-recommended treatment goal for healthy older adults with diabetes (HbA1c < 7.5%) was achieved by 59% of the participants receiving Glar-300 and 58% receiving Glar-100. HbA1c < 7.0% was achieved by 34% and 37% of the participants in the Glar-300 and Glar-100 groups, respectively.

Otherwise, when considering the ≤70 mg/dL threshold, participants were at a lower risk of experiencing ≥1 nocturnal confirmed or severe (RR: 0.79; 95% CI: 0.67 to 0.94) and documented symptomatic (RR: 0.79; 95% CI: 0.65 to 0.97) hypoglycemic event with Glar-300 versus Glar-100. Over the 12 mo study period, severe hypoglycemia was reported by 13 (4.0%) participants in the Glar-300 group and 16 (4.8%) participants in the Glar-100 group. Mean change in body weight from BL to month 12 was 1.1 kg (SD: 4.0) for Glar-300 and 1.3 kg (3.3) for Glar-100.

Daily basal insulin dose increased in both treatment groups over 12 mo of treatment to a mean of 0.83 U/kg (SD: 0.38) and 0.73 U/kg (0.35) in the Glar-300 and Glar-100 groups, respectively. The increase was mainly seen in the first 12 wk and occurred to a greater extent with Glar-300 than with Glar-100 (change from BL to month 12: 0.35 U/kg versus 0.23 U/kg, resp.). Incidence of AEs was 68.8% and 68.4% for Glar-300 and Glar-100 groups, respectively; injection site reactions were experienced by 12 (3.7%) participants in the Glar-300 group and 9 (2.7%) participants in the Glar-100 group [72].

In recent post hoc analyses of patient-level data from the EDITION 2 and 3 trials that determined whether previously reported reductions in hypoglycemia were associated with Glar-300 compared with Glar-100 and impacted by a patient risk category in T2DM, the clinical performance measures based on the Healthcare Effectiveness Data and Information Set (HEDIS) were applied to patient-level data. Participants were stratified as low risk (LR) if patients were <65 years old with no comorbidities derived from HEDIS [HbA1c target < 7.0% (53 mmol/mol)] or as high risk if patients either were ≥65 years old or had one or more HEDIS-defined comorbidities [HbA1c target < 8.0% (64 mmol/mol)]. The primary endpoint was a composite of patients achieving HbA1c target without confirmed or severe hypoglycemia over 6 mo in the different treatment groups.

The mean (SD) change in HbA1c from BL to the 6 mo endpoint in the LR cohort in the EDITION 2 trial was −0.7% (1.03) for the Glar-300 group and −0.6% (1.02) for the Glar-100 group, whereas that in the EDITION 3 trial was −1.3% (1.21) versus −1.5% (1.24) for Glar-300 and Glar-100, respectively. Moreover, in the high-risk cohorts in the EDITION 2 trial, the change was −0.6% (1.29) and −0.6% (0.92) for Glar-300 and Glar-100, respectively, and in the EDITION 3 trial, it was −1.2% (1.16) and −1.2 (1.12) for Glar-300 and Glar-100, respectively. At 6 mo, fewer patients in the low-risk cohort who were treated with Glar-300 (in the EDITION 2 trial) had confirmed or severe hypoglycemia compared with those treated with Glar-100 (68.3% versus 76.4%; *P* = 0.0245); this result was not found in the EDITION 3 trial (43.6% versus 49.5%; *P* = 0.136). In the high-risk cohorts, there was no significant difference in the proportion of individuals experiencing confirmed or severe hypoglycemia when treated with Glar-300 compared with Glar-100 in either study.

The events per patient-year of confirmed or severe hypoglycemia in the EDITION 2 trial (for Glar-300 versus Glar-100) were as follows: low risk: 13.4 versus 17.1 events per patient-year (*P* < 0.0001) and high risk: 16.2 versus 21.2 (*P* < 0.0001). Those in the EDITION 3 trial (for Glar-300 versus Glar-100) were as follows: low risk: 5.13 versus 7.62 events per patient-year (*P* < 0.001) and high risk: 9.78 versus 10.9 events per patient-year (*P* = 0.0664). Moreover, the participants in the low-risk cohort who were treated with Glar-300 had a lower incidence of nocturnal hypoglycemia than those treated with Glar-100 in the EDITION 2 trial (29.8% versus 41.8%, *P* = 0.002), whereas those in the EDITION 3 trial had an incidence of 16.7% versus 21.8% (*P* = 0.107). In the high-risk cohort, patients treated with Glar-300 showed a nonsignificant trend toward lower nocturnal hypoglycemia compared with those treated with Glar-100 [25.3% versus 38.0% (*P* = 0.0678) for the EDITION 2 trial and 24.2% versus 32.7% (*P* = 0.1903) for the EDITION 3 trial]. The rate of nocturnal hypoglycemia in the EDITION 2 trial for both cohorts was significantly lower for those treated with Glar-300 versus Glar-100 (low risk: 1.99 versus 3.62 events per patient-year and high risk: 1.54 versus 3.86 events per patient-year, both *P* < 0.0001); for the EDITION 3 trial, the rate was 1.11 versus 1.21 events per patient-year for low risk (*P* = 0.4245) and 1.82 versus 1.68 events per patient-year for high risk (*P* = 0.5943). The authors concluded that there was a statistically nonsignificant trend of more patients treated with Glar-300 achieving the composite endpoint compared with Glar-100, irrespective of whether they were classed as LR or HR [73].

Another recent post hoc analysis examined whether Glar-300 could provide insulin-naïve patients on OADs with reductions in prior OAD therapy without compromising impact on HbA1c, while preserving the hypoglycemia benefit of Glar-300 versus Glar-100. Patient-level data from the randomized controlled EDITION 3 trial and deidentified data from the Clinformatics real-world claims database were analyzed. After initiating basal insulin, OAD use was reduced in the EDITION 3 study [49% of the patients used two OADs at BL versus 8% continuing at 6 mo (*n* = 875)]. Patients with a reduction in the use of OADs (two OADs to one OAD) versus those who remained on two OADs demonstrated significant and comparable HbA1c reduction 6 mo after initiating either Glar-300 or Glar-100, while preserving the relative protection from hypoglycemia previously demonstrated with Glar-300. Consistent with these results, the real-world data also demonstrated a reduction in OAD use [39% of the patients using two OADs at BL versus 23% at 6 mo (*n* = 6430)] following Glar-300 or Glar-100 initiation, with a lower risk of hypoglycemia with Glar-300 compared with Glar-100. These data suggest that patients treated with Glar-300 could step down OAD use without sacrificing glycemic control and with less hypoglycemia than those treated with Glar-100 [74].

A recent retrospective study (DELIVER 3) examined the performance of Glar-300 in older patients with T2DM in real-world clinical settings focusing on glycemic control and hypoglycemia risk. The Predictive Health Intelligence Environment database (representing 26 integrated healthcare delivery networks) was used to identify T2DM patients aged ≥ 65 years on basal insulin who switched to either Glar-300 or other basal insulins (insulin Glar-100, Det, or Deg) from March 1, 2015, to March 31, 2016. Patients had ≥12 mo of BL data and ≥6 mo of follow-up data. The effect of the cohort on HbA1c reduction, hypoglycemia incidence/event rate, and achievement of HbA1c goal at 6 mo was assessed by generalized linear models or logistic regression models with adjustment for BL characteristics.

The analysis included 468 patients switched to Glar-300 and 1142 patients switched to other basal insulins. The mean age was 71.8 and 73.1 years, and mean BL HbA1c levels were 8.52% and 8.34% for the two cohorts, respectively. Switching to Glar-300 versus other basal insulins led to comparable changes in HbA1c [LS mean difference: −0.09 (*P* = 0.24)], and similar proportions of patients in each cohort achieved HbA1c < 7.0% [OR: 0.798 (95% CI: 0.581 to 1.098; *P* = 0.166)] and <8.0% [OR: 0.967 (95% CI: 0.749 to 1.248; *P* = 0.797)]. Patients switched to Glar-300 were 57% less likely to have hypoglycemia at 6 mo follow-up [OR: 0.432 (95% CI: 0.307 to 0.607, *P* < 0.0001)]. After adjusting for BL characteristics, hypoglycemia event rates were also significantly lower in the Glar-300 cohort [LS mean difference: −4.94 events/100 patients/month (*P* = 0.0002)]. In real-world clinical settings, switching to Glar-300 in older patients with T2DM is associated with significantly lower hypoglycemia risk and similar glycemic control compared to switching to other basal insulins [75].

## 6. Discussion

Overall, throughout the EDITION clinical trial program, the treatment effect [i.e., mean change in HbA1c, percentage of patients that achieved HbA1c, change in laboratory-measured FPG, and change in SMPG from BL to endpoint (month 6 and month 12)] of Glar-300 versus Glar-100 was consistent across tested subgroups defined by BL/screening factors such as age, sex, race, ethnicities, BL BMI, duration of diabetes, HbA1c at screening, and geographical area. Based on the predefined noninferiority margin of 0.4%, the noninferiority of Glar-300 compared with Glar-100 shown as the upper bound of the 95% CI was below 0.4% and met even more stringent criteria with the observed upper bound in all studies below 0.3%, indicating that Glar-300 was convincingly noninferior to Glar-100.

It should be noted that although, in the 6 mo EDITION trials, Glar-300 was found to be noninferior to Glar-100 in reducing HbA1c, the 12 mo data for EDITION 1 showed significantly greater reduction in HbA1c with Glar-300 than with Glar-100. This may be due to the fact that the patterns observed for HbA1c (over a less intensive follow-up of the individuals undergoing therapy) gave rise to an initial worsening of glycemic control in both groups at month 9 that persisted until month 12 with Glar-100, but not with Glar-300.

In the EDITION 3 trial, the mean change in FPG from BL to month 6 was greater with Glar-100; additionally, there was a more gradual decrease in prebreakfast SMPG with Glar-300 (probably the PK and PD characteristics of Glar-300, when attributing a duration of action < 24 h, caused the differences identified with regard to Glar-100). Then, the EDITION JP1 trial at month 6 showed significant differences in favor of Glar-300 with regard to SMPG (8-point SMPG profiles, mean predinner SMPG, predinner to bedtime, and average preinjection SMPG), probably indicating, as was the case in EDITION 3, that the PK and PD characteristics of Glar-300 have a stronger impact on metabolic control, particularly on the SMPG profiles. The EDITION JP2 trial showed a higher increase in SMPG after meals (when compared to the findings in the EDITION 1, 2, and 3 trials) that may be related to the evidence of ethnic differences in the pathophysiological characteristics in T2DM. Hence, Asian people show higher sensitivity to insulin, lower insulin secretion, and less *β*-cell regeneration ability, in contrast to Caucasians. However, these differences in glycemic control among the various populations are modest and consequently should not govern the selection of one insulin versus another for DM patients.

The overall conclusion is then that the glycemic control goals achieved with Glar-300 were similar to those achieved with Glar-100. The risk of experiencing ≥1 confirmed (BG ≤ 70 mg/dL) or severe episode of hypoglycemia (nocturnal and at-any-time hypoglycemia) was significantly reduced in the EDITION trials. Such reduction, however, was not consistent in all trials or in all of the definitions of hypoglycemia. For example, the reduced risk of hypoglycemia was more significant (at month 6) in the EDITION 2 trial versus the EDITION 1 trial (it may be likely that the use of fast-acting analogue insulin in the EDITION 1 trial has impacted the difference found in the reduced risk of hypoglycemia between both trials). Despite the difference shown, it should be highlighted that the number of severe hypoglycemic events in the EDITION 2 trial was rare (just 10 participants in total). The EDITION 3 trial, on the other hand, had low power to identify any differences between treatments, since the number of hypoglycemic events was low, which may account for the reduced risk of nocturnal and at-any-time hypoglycemia. Significant differences were only reached over the BL to month 6 period (nocturnal hypoglycemia).

Another finding was that, throughout the EDITION trials, the goal of reducing the risk of hypoglycemia (at any time) during the follow-up period from week 9 to month 6 was not met. This may be due to the switch of basal insulin that the subjects received previously over to Glar-300. Hence, the results observed during the maintenance phase (for this definition of hypoglycemia) could have been affected by the “learning” process of patients when using a new basal insulin. Nonetheless, the risk of hypoglycemia (nocturnal) over the same follow-up period (week 9 to month 6) was reduced in the EDITION 1, 2, and JP2 trials. The risk of hypoglycemia (at any time) over the BL to month 12 period was only reduced in the EDITION 1 trial, without any significant differences with the other EDITION trials for this definition of hypoglycemia. The risk of nocturnal hypoglycemia ≤ 70 mg/dL was reduced in the EDITION 1 and 2 trials (for all follow-up periods), while in the EDITION 3, 4, JP1, and JP2 trials, the risk was reduced in at least 1 of the follow-up periods (at month 6) and in the EDITION 1, 2, and JP2 trials, it was during the follow-up period from BL to month 12.

The treatment effect was less consistent with regard to the risk of documented symptomatic hypoglycemia < 54 mg/dL (BL to month 6). A significant reduction in the risk of hypoglycemia (at any time) was shown in the EDITION 2 trial, while in the EDITION 3 trial, the risk of hypoglycemia, both at-any-time and nocturnal, was reduced and in the EDITION JP1 trial, the risk of nocturnal hypoglycemia was lowered. No differences in the risk of this definition of hypoglycemia were found in the EDITION 1, 4, and JP2 trials with Glar-300 versus Glar-100. Furthermore, during the BL to month 12 follow-up period, a significant reduction was noted in the risk of nocturnal hypoglycemia in the JP1 trial, as well as a significant difference in the risk reduction with Glar-300 for at-any-time hypoglycemia in the EDITION 3 trial (with no significant differences in the EDITION 1, 2, JP1, and JP2 trials with regard to that risk).

Such differences in the risk of hypoglycemia may be explained at least partially, because when comparing “unit by unit” Glar-300 versus Glar-100, a higher dose of Glar-300 is required for glycemic control at the end of the follow-up. Consequently, in the EDITION trials, the start of Glar-300 or Glar-100 was established based on the previous insulin requirements of the participants (or based on body weight), and these requirements were very similar for the different populations studied. According to the protocol, the insulin dose adjustment was done once a week and in a maximum dose of 6 units. The result was that the participants allocated to the Glar-300 group required longer to achieve metabolic control compared to those allocated to the Glar-100 group (in the treat-to-target insulin regimens).

The relative underdosing of the patients receiving Glar-300 could have impacted the lower risk of hypoglycemia, particularly considering the high threshold defined (BG ≤ 70 mg/dL). This may also explain the lower effect on the reduction of the risk of hypoglycemia when using a more demanding threshold (BG ≤ 54 mg/dL). Consequently, a general statement may claim that the use of Glar-300 significantly reduced the risk of hypoglycemia [particularly nocturnal hypoglycemia (BG ≤ 70 mg/dL)] compared to that of Glar-100 in T2DM and T1DM individuals, with a favorable effect, though less significant, on the risk of hypoglycemia (BG ≤ 70 mg/dL) at any time and on the risk of documented symptomatic hypoglycemia at any time and night (BG ≤ 54 mg/dL). This further proves that the time of administration (morning or evening, in T1DM) makes no difference in the risk of hypoglycemia. These aspects associated with Glar-300 may be explained, at least in part, by the smoother, more even PK and PD profiles and the low within-day variability.

There were weight differences between the patients receiving Glar-300 and Glar-100 (Table 5). The weight gain was statistically lower in the EDITION 2 trial (at month 6 and month 12) and the EDITION 4 trial (at month 6), while a significant weight reduction was noted in the EDITION JP1 trial (at month 6) and the JP2 trial (at month 6 and month 12). However, no significant weight differences were observed in the EDITION 1 (at month 6 and month 12), EDITION 3 (at month 6 and month 12), or EDITION JP1 (at month 12) trials. There may be different reasons for these weight differences, such as the initial insulin doses at the beginning of the trials, which could have contributed to a higher weight gain over time. Moreover, among all the EDITION trials, the highest basal insulin dose that patients received was in the EDITION 1 and 2 trials and the effect on weight showed a nonsignificant increase in the EDITION 1 trial for both insulins, although the increase was less significant in those receiving Glar-300 over the BL to month 6 and BL to month 12 periods. In contrast, in the EDITION 2 trial, over the BL to month 6 and BL to month 12 periods, both treatment groups experienced weight gain. However, the patients receiving Glar-300 experienced a lower weight gain compared to the Glar-100 patients (the difference was statistically significant in favor of Glar-300).

In the EDITION 3 and 4 trials, there was a weight increase in both treatment groups, with a lower weight gain in the Glar-300 group (the difference was statistically significant in the EDITION 4 trial, but not in the EDITION 3 trial). Statistically significant differences were found among the Japanese population in favor of Glar-300 in the EDITION JP1 trial (over the BL to month 6 period) that registered a weight loss in patients receiving Glar-300 but a weight gain in those receiving Glar-100. However, over the follow-up from BL to month 12, both treatment groups experienced some weight gain, with a lower impact of Glar-300 (though not statistically significant). Furthermore, in the EDITION JP2 trial (BL to month 6 and BL to month 12), the participants receiving Glar-300 lost weight compared to those receiving Glar-100, who experienced weight gain; the differences in both groups, over both follow-up periods, were statistically significant.

Considering that the glycemic control was similar with both Glar-300 and Glar-100 (regardless of any other medicines that the participants were taking), then it is unlikely that any therapies other than basal insulin influenced the weight differences identified in the EDITION trials. Similarly, the HbA1c levels were similar for all the EDITION trials, and although other variables such as age, weight, BMI, duration of DM, and FPG were different across the different trials, the weight differences always favored Glar-300. This finding indicates that these variables did not affect the outcomes; it is possible that the differences identified among the Japanese population are due to a lower BMI and the shorter duration of the DM disease, as compared with the rest of the population evaluated, with higher BMI and broader ethnic diversity.

While there is no final explanation about the weight differences identified, the most likely hypothesis is that the weight gain differences (notwithstanding a similar glycemic control between Glar-300 and Glar-100) reflect the lower risk of hypoglycemia shown with Glar-300. In other words, if the risk of hypoglycemia was higher among the Glar-100 patients, it is likely that the approaches to overcome the hypoglycemic events based on additional calorie intake (defensive snacking) have impacted the final result of a higher weight gain among the population receiving Glar-100.

The insulin dose required in both treatment arms increased over the follow-up in every EDITION trial; such increase was higher among the patients receiving Glar-300 (particularly over the first 12 wk of treatment). As a whole, the dose increase was 10–15%, although, in the EDITION 4 trial at month 6, the dose increase was higher (with a percentage difference in basal insulin of +17.5%; at month 6, the Glar-300 group required 6.4 more units of basal insulin compared with the Glar-100 group). On the other hand, the dose increase in the EDITION 3 trial at month 12 was 20%. Such increase in the dose of Glar-300 had no negative impact on weight; on the contrary, the weight gain in this trial was lower among those receiving Glar-300. The reasons for the dose increase are not clear; however, one explanation is that the use of Glar-300 generates a more compact insulin depot in the subcutaneous tissue and this compact insulin depot is more prone to the effect of enzyme inactivation of tissue peptidases (this observation suggests a somewhat lower bioavailability of Glar-300 compared with Glar-100). This leads to the need to increase the total basal insulin dose.

AIA-positive patients in both the Glar-300 and Glar-100 groups already had higher basal insulin doses at BL compared with AIA-negative patients. However, mean changes in Glar-300 and Glar-100 doses from BL to month 6 were similar regardless of the AIA status (positive or negative).

Moreover, across the EDITION trials, Glar-300 was generally well tolerated; the TEAEs, serious TEAEs, TEAEs leading to discontinuation, and drop-out rates were generally low and balanced between groups (the reasons for discontinuation were balanced between groups). Notably, the highest discontinuation rate was observed for “other reasons.” The majority of discontinuations were not safety related; single cases were due to perceived lack of efficacy or hypoglycemia; however, these cases were balanced between groups. The most common AEs were infections, nervous system disorders, gastrointestinal events, cardiac events, and musculoskeletal complaints, which occurred equally in both groups.

The number of casualties throughout the trials was very low (and none was considered medication-related). The good tolerability of Glar-300 and the presence of TEAEs similar to those with Glar-100 may be due to the fact that the metabolism of Glar-300 is the same as that of Glar-100, with the M1 metabolite (21A-Gly-human insulin) being the main active circulating moiety. This may be relevant, since it implies that the neutral safety profile with regard to cardiovascular outcomes and cancer incidence that was demonstrated for Glar-100 in the ORIGIN trial should also be applicable for Glar-300.

Finally, substudies and post hoc analyses (in participants with T2DM) have found that the efficacy and safety of Glar-300 are maintained when the Glar-300 was injected up to 3 h before or after the usual time of administration, with low rates of hypoglycemia and AEs (and similar control in the levels of HbA1c). It was also found that the switching from twice-daily basal insulin to once-daily Glar-300 or Glar-100 led to reductions in HbA1c, with comparable decreases in both treatment groups and a lower risk of nocturnal confirmed or severe hypoglycemia observed with once-daily Glar-300 versus once-daily Glar-100 in participants switching from twice-daily basal insulin. On the other hand, it was also found that Glar-300 provided comparable glycemic control with less hypoglycemia, regardless of age of diabetes onset, age, BMI, diabetes duration, and renal function. The results of these studies demonstrated the efficacy and safety of Glar-300 in special and specific populations.

### 6.1. Evidence Strengths and Limitations

There are several limitations in the EDITION trials, including the fact that the trials were all open-label because Glar-300 and Glar-100 use distinct pen injector designs. This could lead to technology bias in favor of the new insulin or familiarity bias in favor of the comparator; additionally, there is also a concern over possible confounding by adjustment of the prandial insulin dose in the EDITION 1, 4, and JP1 trials. In the EDITION 1 and 2 trials, participants were required to have current basal insulin treatment of ≥42 U/d, and these results may not be generalizable to people with lower basal insulin requirements. More hypoglycemic events in people with T2DM occurred during the day rather than at night in the EDITION 1, 2, and 3 trials (possibly related to prandial rather than basal insulin in the EDITION 1 trial); however, the prolonged action of Glar-300 may have caused a relative shift of long-acting insulin action from night to day. The BL characteristics were largely similar between groups in the EDITION 1, 2, and 3 trials. Except for the Japanese population with T2DM, in the EDITION 1, 2, and 3 trials, the mean BMI was high and results may not be generalizable to people with a lower BMI.

### 6.2. Value of Glar-300 in Clinical Context

A considerable number of people with T1DM or T2DM may benefit from the use of Glar-300, such as people at high risk of hypoglycemia or patients with a high rate of hypoglycemic events while in treatment with Glar-100. Individuals requiring more than one daily dose of basal insulin or people that need some “flexibility” in the time of administration of insulin (which may improve compliance since the rigid treatment schedules are no longer required) may also benefit. Additionally, patients require large doses of basal insulin, since Glar-300 requires a lower total dose volume than Glar-100 (meaning large insulin doses in a smaller volume, administering up to 80 U in one single injection, and theoretically causing less pain at the injection site).

The lower weight gain with Glar-300 may be an additional critical factor to consider when selecting a basal insulin; thus, whenever weight loss is a therapeutic objective in patients receiving insulin, Glar-300 offers a valid option for managing DM. The subgroup analysis of individuals ≥ 65 years showed that switching to Glar-300 (versus switching to other basal insulins) is associated with a significantly lower risk of hypoglycemia, potentially resulting in higher patient satisfaction and better compliance and persistence with therapy. Furthermore, patients with mild-to-moderate renal impairment suggest that Glar-300 can be used in this population with frequent monitoring and dose adjustment. Finally, the highest average dose received with Glar-300 did not affect the efficacy and safety of the medication. Cost, however, is a consideration when increasing the final dose of Glar-300 versus Glar-100.

### 6.3. Areas of Uncertainty and Future Studies

The efficacy and safety of Glar-300 should be studied in special populations (i.e., subjects aged < 18 years, pregnant women, hospitalized patients, and dialysis patients) and compared with those of other basal insulins (i.e., Det, Deg, and concentrated insulin) and of combined therapy at fixed doses with new therapies (i.e., GLP-1 receptor agonists and SGLT2 inhibitor). A further evaluation is needed with regard to endpoints such as mortality and micro- and macrovascular complications with respect to other basal insulins. It should also be established whether the lower risk of hypoglycemia in individuals ≥ 65 years translates into lower healthcare resource utilization.

### 6.4. Conclusions

Throughout the EDITION clinical trial program, the glycemic control goals achieved with Glar-300 were similar to those achieved with Glar-100, with a lower risk of hypoglycemia and less weight gain. These results suggest that Glar-300 may have a place as an alternative, long-acting basal insulin for patients with T1DM or T2DM.

## Figures and Tables

**Table 1:** Baseline demographics and patient characteristics of participants in the EDITION clinical trial program.

Study description and treatment	EDITION 1		EDITION 2		EDITION 3		EDITION 4		EDITION JP1		EDITION JP2	
*Number of participants (mITT)*												
Glar-300	404		403		432		273		122		120	
Glar-100	400		405		430		273		121		120	
*Glucose-lowering therapy at screening (%)*	Glar-300	Glar-100	Glar-300	Glar-100	Glar-300	Glar-100	Glar-300 (N/A)	Glar-100 (N/A)	Glar-300 (N/A)	Glar-100 (N/A)	Glar-300	Glar-100
Prior use of metformin	56.2	58.6	94.8	93.6	90.6	92.0					57.9	59.2
Prior use of SU	0.2	0.0	2.0	0.5	59.1	58.6					52.9	55
Prior use of DPP-4 inh.	0.0	0.0	7.7	12.5	20.7	22.4					42.1	44.2
Prior use of GLP-1 agonist	0.0	0.0	0.0	0.7	0.0	0.5					EC	EC
Prior use of thiazolidinediones	1.5	3.4	1.5	3.4	NR	NR					9.9	7.5
Prior use of *α*-glucosidase inh.	NR	NR	1.0	0.2	NR	NR					35.5	21.7
Prior use of glinides	NR	NR	NR	NR	NR	NR					9.1	10.0
Combinations of OADs	NR	NR	1.2	2.5	NR	NR					NR	NR
*Predominant ethnic group*	Caucasian		Caucasian		Caucasian		Caucasian		Asian (Japanese)		Asian (Japanese)	
*Prior use of insulin glargine (%)*	92.3	91.6	74.9	82.8	N/A		85.0	78.5	90.2	90.1	98.3	91.7
*Inclusion criteria*												
HbA1c (%)	≥7.0, ≤10.0		≥7.0, ≤10.0		≥7.0, ≤11.0		≥7.0, ≤10.0		≥7.0, ≤10.0		≥7.0, ≤10.0	
Age (years)	≥18		≥18		≥18		≥18		≥18		≥18	
*Mean baseline characteristics*	Glar-300	Glar-100	Glar-300	Glar-100	Glar-300	Glar-100	Glar-300	Glar-100	Glar-300	Glar-100	Glar-300	Glar-100
Age, years (mean)	60.1	59.8	57.9	58.5	58.2	57.2	46.4	48.2	44.1	46.3	61.1	60.5
Weight (kg)	106.2	106.4	98.7	98	95.1	95.6	81.9	81.8	63.9	61.0	67.4	65.9
BMI (kg/m^2^)	36.6	36.6	34.8	34.8	32.8	33.2	27.6	27.6	23.8	23.2	25.7	24.8
Duration of DM (years)	15.6	16.1	12.7	12.5	10.1	9.6	20.5	21.4	12.2	13.9	14.0	13.9
HbA1c (%)	8.15	8.16	8.26	8.22	8.51	8.57	8.11	8.14	8.06	8.07	7.99	8.06
FPG (mg/dL)	158.3	160.7	148.3	142	178.7	183.8	185.9	199.3	187	183	138.7	133.3
Basal insulin dose (U/kg/day)	0.67	0.67	0.66	0.68	N/A	N/A	0.38	0.37	0.28	0.30	0.25	0.24
Mealtime insulin dose (U/kg/day)	0.54	0.54	N/A	N/A	N/A	N/A	0.34	0.33	0.45	0.45	N/A	N/A
Preinjection SMPG, mean (mg/dL)	185.7	188.1	198.3	195.2	196.9	202.3	177.6	186.3	177.6	178.6	176.8	178.1

*α*-glucosidase inh.: alpha-glucosidase inhibitors; BMI: body mass index; DM: diabetes mellitus; DPP-4 inh.: inhibitors of dipeptidyl peptidase 4; EC: exclusion criteria; FPG: fasting plasma glucose; Glar-100: insulin glargine 100 U/mL; Glar-300: insulin glargine 300 U/mL; GLP-1 agonist: glucagon-like peptide-1 receptor agonists; mITT: modified intention to treat; N/A: not applicable; NR: not reported; OADs: oral antihyperglycemic drugs; SU: sulphonylurea.

**Table 2:** Main glycemic responses of the participants in the EDITION clinical trial program.

Glycemic responses	EDITION 1		EDITION 2		EDITION 3		EDITION 4		EDITION JP1		EDITION JP2	
	Glar-300	Glar-100	Glar-300	Glar-100	Glar-300	Glar-100	Glar-300	Glar-100	Glar-300	Glar-100	Glar-300	Glar-100
Mean change in HbA1c (%) from BL at month 6	−0.83	−0.83	−0.57	−0.56	−1.42	−1.46	−0.42	−0.44	−0.3	−0.43	−0.45	−0.55
Mean change (difference) in HbA1c (%) from BL at month 6	−0.00 (95% CI: −0.11 to 0.11)		−0.01 (95% CI: −0.14 to 0.12)		0.04 (95% CI: −0.09 to 0.17)		0.04 (95% CI: −0.10 to 0.19)		0.13 (95% CI: −0.03 to 0.29)		0.10 (95% CI: −0.08 to 0.27)	
% of patients achieved HbA1c target (<7.0%) at month 6	39.6	40.9	30.6	30.4	43.1	42.1	16.8	15	15.6	20	25	24
Mean change in HbA1c (%) from BL at month 12	−0.86	−0.69	−0.55	−0.50	−1.29	−1.21	−0.20	−0.22	−0.2	−0.3	−0.3	−0.3
Mean change (difference) in HbA1c (%) between BL and month 12	−0.17 (95% CI: −0.30 to −0.05)		−0.06 (95% CI: −0.22 to 0.10)		−0.08 (95% CI: −0.23 to 0.07)		0.02 (95% CI: −0.13 to 0.17)		0.2 (*P* = NS)		0.0 (95% CI: −0.2 to 0.2)	
Change in laboratory-measured FPG (mmol/L) between BL and month 6	−1.48	−1.69	−1.14	−1.06	−3.41	−3.80	−0.42	−1.45	−0.75	−1.2	−1.21	−1.25
Change in laboratory-measured FPG (mmol/L) between BL and month 12	−1.6	−1.4	−0.8	−1.0	−3.16	−3.23	−0.43	−1.39	−0.8	−0.4	−0.7	−1.0
Change in preinjection SMPG between BL and month 6 (mmol/L), LS mean change	−0.90	−0.84	−0.78	−0.57	−2.16	−2.33	−1.17	−0.82	−0.60	0.40	0.70	0.90

BL: baseline; FPG: fasting plasma glucose; Glar-100: insulin glargine 100 U/mL; Glar-300: insulin glargine 300 U/mL; LS: least squares; NS: not significant; SMPG: self-monitored plasma glucose.

**(a):** 

Study 6 mo treatment period	Relative risk of experiencing ≥1 confirmed (blood glucose ≤ 70 mg/dL) or severe episode of hypoglycemia (95% CI)						Relative risk (95% CI) of documented symptomatic hypoglycemia (<54 mg/dL)	
	Nocturnal (00:00–05:59 h)			At any time (24 h)			At any time	Nocturnal
	BL to W8	W9 to month 6	BL to month 6	BL to W8	W9 to month 6	BL to month 6	BL to month 6	BL to month 6
EDITION 1	0.79 (0.64 to 0.98)	0.79 (0.67 to 0.93)	0.78 (0.68 to 0.89)	0.86 (0.78 to 0.94)	0.96 (0.89 to 1.04)	0.93 (0.88 to 0.99)	0.90 (0.76 to 1.07)	0.72 (0.51 to 1.01)
EDITION 2	0.53 (0.39 to 0.72)	0.77 (0.60 to 0.97)	0.71 (0.58 to 0.86)	0.78 (0.69 to 0.89)	0.91 (0.82 to 1.02)	0.90 (0.83 to 0.98)	0.77 (0.60 to 0.98)	0.71 (0.46 to 1.08)
EDITION 3	0.74 (0.48 to 1.13)	0.90 (0.67 to 1.22)	0.76 (0.59 to 0.99)	0.83 (0.67 to 1.03)	0.86 (0.74 to 1.00)	0.88 (0.77 to 1.01)	0.55 (0.37 to 0.82)	0.51 (0.27 to 0.94)
EDITION 4	0.82 (0.70 to 0.96)	1.06 (0.92 to 1.23)	0.98 (0.88 to 1.09)	0.98 (0.92 to 1.04)	0.98 (0.91 to 1.06)	1.00 (0.95 to 1.04)	0.99 (0.88 to 1.11)	1.05 (0.86 to 1.29)
EDITION JP1	0.71 (0.56 to 0.91)	0.84 (0.70 to 1.00)	0.85 (0.73 to 0.99)	0.91 (0.84 to 0.99)	1.01 (0.95 to 1.08)	0.99 (0.95 to 1.04)	0.87 (0.75 to 1.00)	0.64 (0.47 to 0.88)
EDITION JP2	0.83 (0.45 to 1.52)	0.58 (0.40 to 0.85)	0.62 (0.44 to 0.88)	0.69 (0.52 to 0.91)	0.84 (0.70 to 1.01)	0.86 (0.73 to 1.01)	1.44 (0.67 to 3.10)	1.17 (0.48 to 2.84)

**(b):** 

Study 12 mo treatment period	Relative risk of experiencing ≥1 confirmed (blood glucose ≤ 70 mg/dL) or severe episode of hypoglycemia (95% CI)		Relative risk (95% CI) of documented symptomatic hypoglycemia (<54 mg/dL)	
	Nocturnal (00:00–05:59 h) BL to month 12	At any time (24 h) BL to month 12	BL to month 12	BL to month 12
EDITION 1	0.84 (0.75 to 0.94)	0.94 (0.89 to 0.99)	0.91 (0.79 to 1.05)	0.87 (0.66 to 1.13)
EDITION 2	0.84 (0.71 to 0.99)	0.96 (0.89 to 1.02)	0.92 (0.74 to 1.15)	0.95 (0.67 to 1.36)
EDITION 3	0.86 (0.69 to 1.07)	0.92 (0.82 to 1.03)	0.65 (0.47 to 0.90)	0.76 (0.48 to 1.22)
EDITION 4	0.97 (0.88 to 1.08)	1.00 (0.96 to 1.05)	0.98 (0.89 to 1.09)	1.00 (0.84 to 1.18)
EDITION JP1	0.93 (0.83 to 1.05)	1.00 (0.96 to 1.04)	0.93 (0.85 to 1.01)	0.79 (0.64 to 0.98)
EDITION JP2	0.73 (0.55 to 0.97)	0.97 (0.85 to 1.10)	1.03 (0.57 to 1.87)	1.14 (0.51 to 2.55)

BL: baseline; Glar-100: insulin glargine 100 U/mL; Glar-300: insulin glargine 300 U/mL; T1DM: type 1 diabetes mellitus; T2DM: type 2 diabetes mellitus; W8: week 8; W9: week 9.

**Table 4:** Proportion of patients with TEAEs, drop-out rates, and rescue therapy percentage in the EDITION trials (6 mo treatment period).

Proportion of patients (%)	EDITION 1		EDITION 2		EDITION 3		EDITION 4		EDITION JP1		EDITION JP2	
	Glar-300	Glar-100	Glar-300	Glar-100	Glar-300	Glar-100	Glar-300	Glar-100	Glar-300	Glar-100	Glar-300	Glar-100
TEAEs	56.4	54.2	58.8	50.7	56.8	55.9	60.9	58.2	62.3	64.5	58.3	56.7
Serious TEAEs	6.4	5.2	3.7	3.7	5.5	5.9	6.2	8.0	2.5	2.5	4.2	3.3
TEAEs leading to treatment discontinuation	1.5	1.7	1.5	1.0	1.1	1.1	1.1	1.1	0.8	0	2.5	0.8
Drop-out rates %^∗^	7.4	7.7	8.9	9.3	14	17	15.7	14.2	4.1	3.3	5.0	1.7
Rescue therapy (%)	NA	NA	5.7	4.9	3.0	2.0	NA	NA	NA	NA	1.66	0.0
Deaths (*n*)^∗∗^	1	2	2	0	1	0	1	0	0	0	0	0

Glar-100: insulin glargine 100 U/mL; Glar-300: insulin glargine 300 U/mL; mITT: modified intention to treat; TEAEs: treatment-emergent adverse events. ^∗^Modified intention-to-treat population; ^∗∗^none of the deaths were considered to be related to the study drug; in the EDITION 1 and 3 trials, additional deaths occurred after treatment discontinuation.

**Table 5:** Change in weight for Glar-300 versus Glar-100 across the EDITION phase 3 clinical trial program.

Study	Change in body weight	*P* value
EDITION 1	There was an increase in body weight in both treatment groups from a baseline (mean ± SD) of 106.11 ± 21.43 kg to 107.04 ± 21.86 kg with Glar-300 and 106.5 ± 19.94 kg to 107.4 ± 20.33 kg with Glar-100. The change was +0.93 kg with Glar-300 and +0.90 kg with Glar-100.	*P* = NS
EDITION 1, 12 mo treatment period	The mean (SD) change was +1.2 kg (3.8) with Glar-300 and +1.4 kg (3.5) with Glar-100 [LS mean difference: −0.2 kg (95% CI: −0.7 to 0.3)].	*P* = NS
EDITION 2	The mean (SD) change was +0.08 kg (3.45) with Glar-300 and +0.66 kg (3.01) with Glar-100.	*P* = 0.015
EDITION 2, 12 mo treatment period	The mean (SD) change was +0.4 kg (4.1) with Glar-300 and +1.2 kg (3.6) with Glar-100 [LS mean difference: −0.7 kg (95% CI: −1.3 to− 0.2)].	*P* = 0.009
EDITION 3	The LS mean change was +0.49 kg (95% CI: 0.14 to 0.83) with Glar-300 and +0.71 kg (95% CI: 0.36 to 1.06) with Glar-100.	*P* = NS
EDITION 3, 12 mo treatment period	The mean (SD) change was +0.97 kg (4.32) with Glar-300 and 1.2 kg (4.16) with Glar-100 [LS mean difference: −0.24 kg (95% CI: −0.81 to 0.33)].	*P* = NS
EDITION 4	The LS mean change (SE) was +0.5 kg (3.3) with Glar-300 and +1.0 kg (3.2) with Glar-100 [LS mean difference: −0.6 kg (95% CI: −1.1 to −0.03)].	*P* = 0.037
EDITION 4, 12 mo treatment period	The LS mean difference in change from BL to month 12 was −0.5 kg (−1.1 to 0.1).	*P* = 0.098
EDITION JP1	The LS mean change (SE) was −0.1 kg (0.2) with Glar-300 and +0.4 kg (0.2) with Glar-100 [LS mean difference: −0.6 kg (95% CI: −1.1 to −0.0)].	*P* = 0.035
EDITION JP1, 12 mo treatment period	The mean change (SE) was +0.06 kg (0.21) with Glar-300 and +0.41 (0.19) with Glar-100.	*P* = NS
EDITION JP2	The LS mean change (SE) was −0.6 kg (0.2) with Glar-300 and +0.4 kg (0.2) with Glar-100 [LS mean difference: −1.0 kg (−1.5 to −0.5)].	*P* = 0.0003
EDITION JP2, 12 mo treatment period	The mean change (SD) was −0.7 kg (0.2) with Glar-300 and +0.5 kg (0.2) with Glar-100.	*P* = 0.0001

Glar-100: insulin glargine 100 U/mL; Glar-300: insulin glargine 300 U/mL; LS: least squares; NS: not significant; SD: standard deviation; SE: standard error.
